# MicroRNA‐382 Is Involved in Acute Kidney Injury via Regulating STAT1 Signaling

**DOI:** 10.1155/jimr/5266272

**Published:** 2026-01-09

**Authors:** Xiaoyan Wang, Guo Cheng, Ting Ren, Zhouping Zou, Xinni Zhang, Shuan Zhao, Siyu Bao, Yingxiang Li, Ping Jia, Yi Fang, Xiaoqiang Ding

**Affiliations:** ^1^ Department of Nephrology, Zhongshan Hospital, Fudan University, Shanghai, China, zs-hospital.sh.cn; ^2^ International Medical Center, Zhongshan Hospital, Fudan University, Shanghai, China, zs-hospital.sh.cn; ^3^ Clinical Research Unit, Institute of Clinical Science, Zhongshan Hospital, Fudan University, Shanghai, China, zs-hospital.sh.cn; ^4^ Shanghai Medical Center of Kidney, Shanghai, China; ^5^ Kidney and Dialysis Institute of Shanghai, Shanghai, China; ^6^ Kidney and Blood Purification Laboratory of Shanghai, Shanghai, China; ^7^ Hemodialysis Quality Control Center of Shanghai, Shanghai, China

**Keywords:** acute kidney injury, macrophages, microRNA, tubular epithelial cells

## Abstract

Acute kidney injury (AKI), characterized by inflammation and oxidative stress, is a life‐threatening clinical presentation. We previously demonstrated the role of miR‐382 in the progression of chronic kidney disease (CKD). However, the role of miR‐382 in AKI is unknown. Genetic depletion of miR‐382 exacerbated renal dysfunction, histologic lesions, apoptosis of epithelial cells, and oxidative stress in kidneys in ischemia‐reperfusion (I/R)‐ and sepsis‐induced AKI. In vitro, downregulation of miR‐382 accompanied with activation of phosphorylation of signal transducer and activator of transcription 1 (STAT1) were induced in mouse renal tubular epithelial cells (mTECs) with hypoxia/reoxygenation (H/R) and lipopolysaccharide (LPS) treatments. Colocalization of phosphorylated STAT1 (p‐STAT1) and *Lotus tetragonolobus* lectin (LTL) suggested the activation of STAT1 signaling in renal epithelial after I/R injury (IRI). Overexpression of miR‐382 in mTECs abrogated phosphorylation of STAT1, generation of interleukin‐6 (*Il-6*), tumor necrosis factor‐*α* (*Tnf-α*), and *Il-1β* as well as reactive oxygen species (ROS) and apoptosis after H/R‐ and LPS treatments. Additionally, loss of miR‐382 was found in isolated macrophages from kidney during I/R‐induced AKI. Upregulation of miR‐382 in Raw264.7 cells inactivated STAT1 signaling and hindered polarization of M1 macrophages inhibiting *Il-6* and inducible nitric oxide synthase (*iNos*). Inhibition of STAT1 pharmacologically reduced *Il-1β*, *Il-6*, and *Tnf-α* in Raw264.7 after H/R. Taken together, these results demonstrated the protective role of miR‐382 in I/R‐ and sepsis‐induced AKI, suggesting miR‐382 as a novel therapeutic strategy for AKI.

## 1. Introduction

Acute kidney injury (AKI), characterized by decline in renal function rapidly, suffers in 15% of hospitalized patients and even 50% of patients in intensive care units (ICUs) [1]. Renal ischemia‐reperfusion (I/R), sepsis, and nephrotoxins, such as the chemotherapeutic agent cisplatin, are the common causes of AKI. The I/R injury (IRI) of kidney is common during surgery as restriction and restoration of blood supply to kidneys [2]. Sepsis‐induced AKI causes high mortality rates in ICU featured as hyperinflammation and multiorgan dysfunction as it is a systemic response to severe infections [3]. Although therapy strategies of AKI get significant progression, high morbidity and mortality of AKI is still unavoidable and effective targets remain to be explored [1]. As previously reported, reactive oxygen species (ROS)‐mediated injury plays an important role in AKI [4]. Renal proximal tubular epithelial cells (PTECs) are susceptible to lesion of ROS production, which subsequently contributes to inflammation, apoptosis, and necrosis of TECs [5]. Therefore, ROS may be a promising therapeutic target for AKI. Additionally, macrophages play a crucial role in modulation of inflammation [6]. Sustained accumulation and activation of immune cells in kidneys extend periods of ischemia due to vascular congestion and induce tubular cell lesion due to cytokine storm [7].

MicroRNAs (miRNAs), endogenous, small noncoding RNAs, generally function as negative regulators of gene expression at the post‐transcriptional level. Increasing evidence shows that miRNAs play an important role in several pathologic processes in AKI. For example, the recently demonstrated roles of regulation of apoptosis and inflammation was found in a typical miRNA miR‐21 during AKI [8]. As reported, miR‐126 protects against renal IRI by promoting vascular integrity after AKI [9]. And miR‐34a was also demonstrated to maintain cell survival during cisplatin induced‐AKI [10]. MiR‐10a ameliorates diabetic nephropathy via negatively regulated inflammation [11]. MiR‐20a attenuates renal IRI by inhibiting ferroptosis [12]. Our previous study showed that miR‐382 was a critical mediator of epithelial–mesenchymal transition, which contributes to renal fibrosis in chronic kidney disease (CKD) [13, 14]. However, the specific roles of miR‐382 in IRI‐ or sepsis‐induced AKI remain unclear.

The signal transducer and activator of transcription (STAT) family, including several proteins, regulates downstream by binding DNA in response to various extracellular cytokines and growth factors. The STAT family of proteins contain SH2 and SH3 domains which specifically bind peptides containing phosphorylated tyrosine. When phosphorylated, STAT proteins form homo‐ or heterodimers, translocate into the nucleus and bind to specific recognition motifs in the promoter regions of target genes, which finally regulate gene’s transcriptional expression. The STAT signaling would be involved in pathologies of AKI as it was reported that ROS could activate JAK‐STAT pathway and ROS‐induced injury plays a critical role in AKI [15, 16]. STAT1 is a well‐characterized proinflammatory signaling molecule extensively implicated in the pathogenesis of AKI [17–19]. Furthermore, the reciprocal suppression relationship between miR‐382 and STAT1 has been implicated in pancreatic cancer and bronchopulmonary dysplasia (BPD) [20, 21]. However, the role of the miR‐382/STAT1 pathway in AKI remains unclear.

Thus, in this study, we aimed to assess the roles of miR‐382 in hypoxia/reoxygenation (H/R) and lipopolysaccharide (LPS) induced AKI, elucidating by apoptosis, inflammation, cell viability, and ROS generation in tubular epithelial as well as polarization of inflammatory macrophages.

## 2. Materials and Methods

### 2.1. Animal Models of AKI

Male C57BL/6J mice were obtained from the Animal Resource Center of Zhongshan Hospital (Fudan University, Shanghai, China), and miR‐382^−/−^ mice were bred from C57BL/6 J mice back‐crossed to miR‐382^−/−^ mice (Bioray Laboratories, Shanghai, China). Our animal experiments were approved by the Animal Care and Use Committee of Zhongshan Hospital and performed according to the National Institutes of Health Guide for the Care and Use of Laboratory Animals. 8–10‐week‐old mice (20–25 g) were used for AKI models. Bilateral renal pedicle clamping for 30 min at 36.5–37°C was induced as IR model and the same surgical procedure except for renal pedicle clamping was performed in sham mice, as previously described [22]. A single intraperitoneal injection of 8 mg/kg LPS from the *Escherichia coli* strain 0111:B4 (Sigma, Shanghai, China) was performed in sepsis‐induced AKI and the same dosage of saline as control administration [23]. For both models, each group included six mice.

### 2.2. Preparation of Single‐Cell Suspensions of Kidney Cells

Kidneys were removed and kept in cold 1× phosphate‐buffered saline (PBS) and then minced into smaller pieces and digested with 1 mg/mL collagenase (catalog no. 17,018‐029; Gibco, NY, USA) in PBS in a gentleMACS Octo dissociator with heaters (Miltenyi Biotec, Germany), followed by filtration through a 70‐μm mesh filter to obtain single‐cell suspensions. Samples were prepared for flow cytometry staining.

### 2.3. Kidney Macrophage Isolation by Flow Cytometry

I/R induced‐AKI models were performed as described above. Single‐cell suspensions of mouse kidney from I/R for 6 and 24 h or sham operation were obtained as described above. All the samples were incubated with an antibody to block Fc receptor, followed by APC/Cyanine7‐conjugated anti‐CD45, FITC‐conjugated anti‐CD11b, and PE‐conjugated anti‐F4/80 antibodies. The CD45^+^CD11b^+^F4/80^+^ cells were sorted and purified via flow cytometry (BD FACS Aria II; BD Biosciences, Shanghai, China), and total RNA was extracted from the sorted CD45^+^CD11b^+^F4/80^+^ cells for reverse transcription‐quantitative polymerase chain reaction (RT‐qPCR) assay.

### 2.4. Cell Culture and Transfection

Mouse renal tubular epithelial cells (mTECs) were a kind gift from the Urology Department of Zhongshan Hospital in Fudan University. Raw264.7 cells were purchased from the American Type Culture Collection. Cells were cultured in Dulbecco’s Modified Eagle medium with high glucose (catalog no. 11,965,092; Gibco) supplemented with 10% fetal bovine serum (catalog no. 10,099,141; Gibco) and 1% penicillin/streptomycin (catalog no. KGY0023; Keygen). H/R model was induced by a hypoxia incubator with 1% O_2_ for 24 h, followed by incubation in normoxic conditions (21% O_2_) for 1 h in mTECs. Cells were treated with 5 μg/mL LPS for 24 h in mTECs for LPS model. Raw264.7 cells were treated with 1 μg/mL of LPS for 24 h to obtain M1 macrophages or with 50 ng/mL of interleukin (*Il*)‐4 for 48 h to obtain M2 macrophages. Knockdown of miR‐382 was achieved by transfecting cultures with 100 nM of locked nucleic acid‐modified anti‐miR‐382 (Exiqon, Shanghai, China). Overexpression of miR‐382 was achieved by transfecting cultures with 100 nM of an miR‐382 mimic (Exiqon, Shanghai, China) for 24 h before subsequent treatments. Scramble miRNA mimic and negative control (NC) were used as transfection controls. The inhibition of STAT1 was achieved by treating cultures with 5 μM fludarabine (catalog no. HY‐B0069; MCE, USA) 12 h before other treatments.

### 2.5. Quantification of Serum Creatinine Levels

Serum creatinine levels were quantified in 30 μL of mouse serum using a QuantiChrom creatinine assay kit (BioAssay Systems, Hayward, CA, USA) according to the manufacturer’s instructions.

### 2.6. ROS Detection

ROS generation in vitro was measured using the ROS Assay Kit (Beyotime, Shanghai, China). ROS production in vivo was detected in frozen kidney sections stained with dihydroethidium. Images were acquired using an Olympus FV1000 confocal microscope (Zhongshan hospital, Shanghai, China). Mean fluorescence intensity was calculated from five random areas per sample using ImageJ (National Institutes of Health).

### 2.7. TUNEL Assay

Apoptosis was detected in mTECs from Caltag Medsystems (Buckingham, UK) and frozen kidney sections using the TUNEL apoptosis detection kit (Beyotime, Shanghai, China). Nuclei were stained with Hoechst33258. The number of TUNEL^+^ cells were counted in five random areas per sample in images obtained with the Olympus FV1000 confocal microscope.

### 2.8. Immunofluorescence

Kidneys were fixed with 10% formalin, embedded in paraffin wax, and sliced into 4 μm. The sections were dewaxed and permeabilized with 0.5% Triton X‐100 for 10 min, followed by blocking with 5% bovine serum albumin for 1 h at room temperature. Next, the sections were incubated with a primary antibody against phosphorylated STAT1 (p‐STAT1; ab109461; Abcam, USA) overnight at 4°C, washed three times with 1× PBS, 10 min each, and incubated with the secondary Cy3‐conjugated donkey anti‐rabbit IgG antibody for 30 min at 37°C. NC antibody staining for p‐STAT1 Ser727 in renal sections and Raw264.7 were performed (Figure S7). Proximal tubules were stained with an anti‐*Lotus tetragonolobus* lectin (LTL) antibody for 2 h at room temperature. Nuclei were stained with Hoechst33258 for 5 min at room temperature, and the slides were washed three times with 1× PBS, 10 min each. Images were acquired using the Olympus FV1000 confocal microscope. Cell slides were fixed in 4% paraformaldehyde for 15 min before following the protocol described for renal sections.

### 2.9. Fluorescent miRNA In Situ Hybridization

Mmu‐miR‐382‐5p in situ hybridization was performed using a customized kit according to the manufacturer’s instructions as previously reported [14].

### 2.10. Histologic Evaluation and Immunohistochemistry

Kidneys were fixed with 10% formalin, embedded in paraffin wax, and sliced into 4‐μm‐thick sections for hematoxylin/eosin staining. Immunohistochemical staining was performed as previously described [13]. Briefly, an anti‐F4/80 primary antibody (catalog no. 70,076; Cell Signaling Technology) was used with a horseradish peroxidase‐conjugated goat anti‐rabbit IgG secondary antibody (catalog no. ZB‐2301; Zhong Shan Gold Bridge Biotechnology, China). The sections were evaluated using a Leica DM 6000B microscope (200x magnification; Leica Microsystems, Wetzeler, Germany).

### 2.11. Immunoblotting

Proteins were extracted from mTECs, Raw264.7 cells, and kidney tissue, as previously described [2]. Primary antibodies against p‐STAT1 S727 (ab109461, Abcam), STAT1 (ab210524, Abcam), and *β*‐actin (GeneTex) were used with peroxidase‐conjugated AffiniPure goat anti‐rabbit IgG (H + L) secondary antibody (111‐035‐003, Jackson ImmunoResearch). Protein levels were quantified using the Image Lab software, version 3.0 (Bio‐Rad, Hercules, CA, USA)

### 2.12. Real‐Time RT‐qPCR

Total RNA from PTECs, Raw264.7 cells, and kidney tissue were extracted using Trizol and reverse transcribed into cDNA using the PrimeScript RT reagent kit.*β*‐Actin was used for normalization. Expression levels of *miR-382* were measured with Taqman probes, and *U6* was used to normalize miR‐382 expression. The following primer sequences were used for RT‐qPCR: tumor necrosis factor (*Tnf)-α* forward: CCCTCACACTCAGATCATCTTCT, reverse: GCTACGACGTGGGCTACAG; *Il-1β* forward: CTGTGACTCATGGGATGATGATG, reverse: CGGAGCCTGTAGTGCAGTTG; *Il-6* forward: TAGTCCTTCCTACCCCAATTTCC, reverse: TTGGTCCTTAGCCACTCCTTC; inducible nitric oxide synthase (*iNos*) forward: CAGATCGAGCCCTGGAAGAC, reverse: CTGGTCCATGCAGACAACCT; *Il-10* forward: GCTCTTACTGACTGGCATGAG, reverse: CGCAGCTCTAGGAGCATGTG; neutrophil gelatinase‐associated lipocalin (*Ngal*) forward: TGGCCCTGAGTGTCATGTG, reverse: CTCTTGTAGCTCATAGATGGTGC. *miR-382* gene expression forward: CCCCACCTCACTAACACTC; reverse: ACATCCATACTTGGCTTCTC

### 2.13. Determination of Cell Viability

Cell viability was determined using the cell counting kit‐8 (CCK‐8) assay. Briefly, PTECs were cultured in 96‐well plates at a density of 2 × 10^3^ per well. At the end of treatments, the CCK‐8 reagent was added to the medium, and the cultures were incubated for 1–4 h at 37°C. Optical density at 450 nm was measured using a microplate reader, and cell viability was calculated according to the manufacturer’s protocol.

### 2.14. Statistical Analysis

All in vivo and in vitro experiments were performed in biological replicates. All the experiments were replicated at least twice. Data were expressed as means ± standard error of the mean. Data are presented as mean ± SEM. The differences between the two groups were evaluated using two‐tailed, unpaired *t*‐tests. For multiple comparisons, one‐way ANOVA followed by Bonferroni post hoc test was employed. A *p*‐value less than 0.05 was considered statistically significant.

## 3. Results

### 3.1. Expression of miR‐382 in I/R and LPS‐Induced AKI Models

First, we evaluated the abundance of miR‐382 in kidneys after AKI using the IRI and sepsis models. As shown in Figure 1a,b, miR‐382 levels were significantly reduced in renal tissue at 6, 12, and 24 h after IRI and LPS treatment. Similarly, the expression levels of miR‐382 were significantly reduced in mTECs exposed to H/R for 2, 4, 6, and 24 h (Figure 1c) or to LPS for 3, 6, 12, and 24 h (Figure 1d).

Figure 1Abundance of miR‐382 in I/R and LPS induced acute kidney injury models. (a) AKI was performed by I/R (30 min) for 6, 12, and 24 h and sham operation as control. Abundance of miR‐382 decreased significantly in kidney of I/R. (b) AKI was performed by LPS with dosage 8 mg/kg for 6, 12, and 24 h, NS as control. Level of miR‐382 was inhibited notably in renal of LPS treatment. (c) Abundance of miR‐382 was decreased in mTECs after hypoxia/reoxygenation (H/R) treatment for 2, 4, 6, and 24 h. U6 served as a standard for normalization. (d) MiR‐382 declined in mTECs challenged with LPS (5 μg/mL) for 3, 6, 12, and 24 h, compared with control group. (e) M1 macrophages were activated with LPS (1 μg/mL LPS for 24 h) and M2 macrophages were activated with *Il*‐4 (50 ng/mL *Il*‐4 for 48 h) in Raw264.7, respectively. Level of miR‐382 decreased in M1 macrophages while increased in M2 macrophages. (f) AKI was performed with IRI model and renal macrophages were identified as CD45^+^F4/80^+^CD11b^+^ cells and were isolated from mice kidneys via flow cytometry. Compared with sham operation, miR‐382 was inhibited significantly in sorted macrophages after IRI for 6 and 24 h. Data are presented as means ± SEM; *N* = 4.  ^∗^ *p* < 0.05;  ^∗∗^ *p* < 0.01. The experiments were replicated at least twice.

**Figure:**
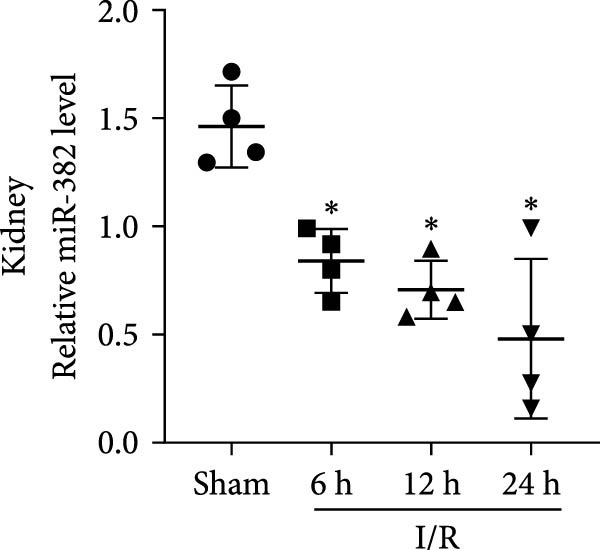
(a)

**Figure:**
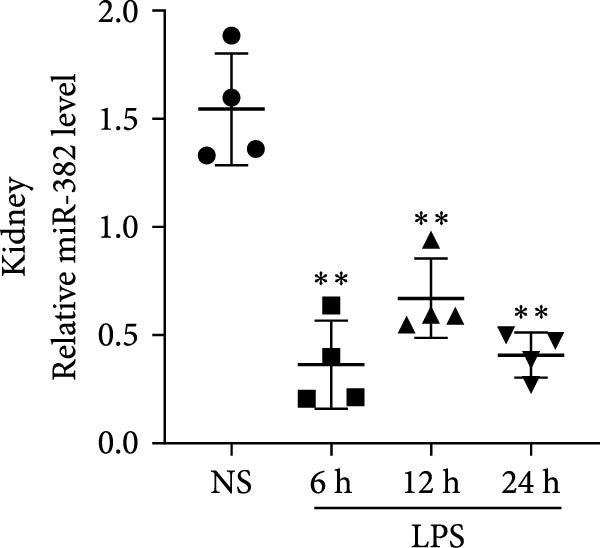
(b)

**Figure:**
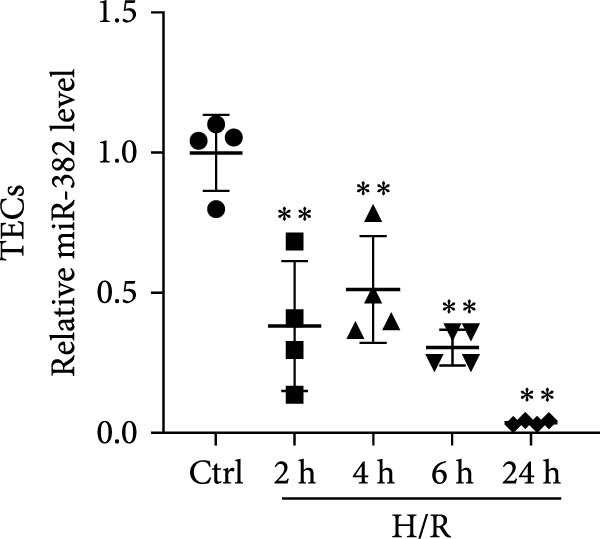
(c)

**Figure:**
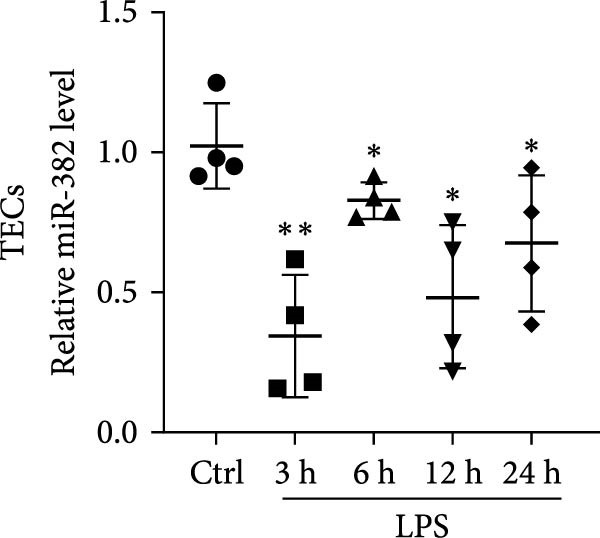
(d)

**Figure:**
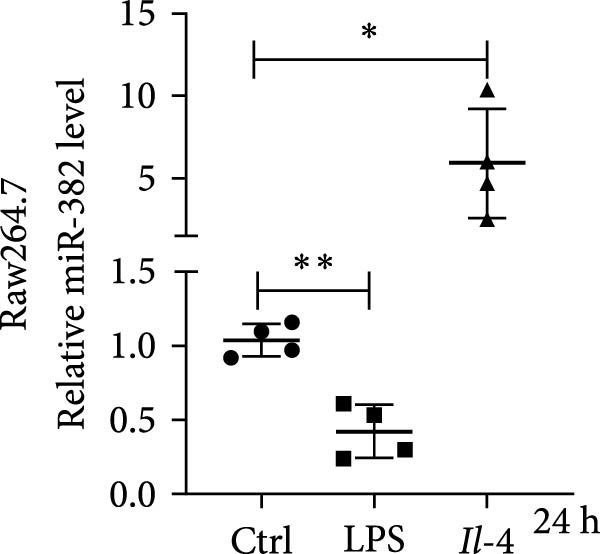
(e)

**Figure:**
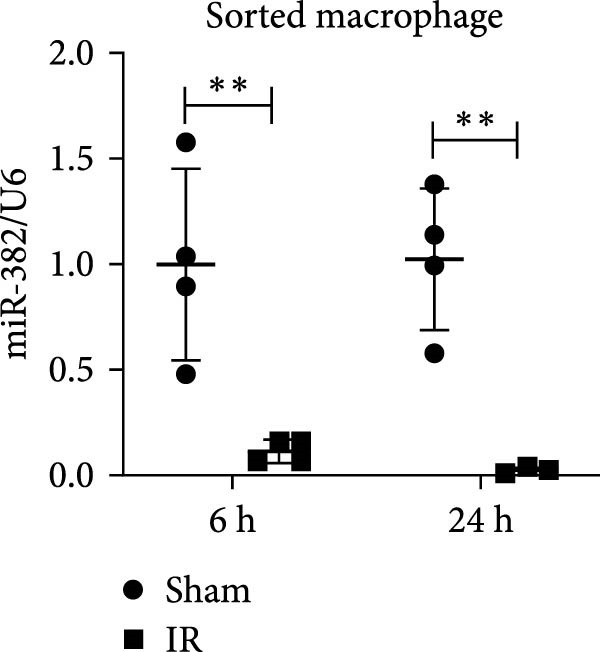
(f)

Next, we evaluated miR‐382 expression in macrophages. To that end, we activated Raw264.7 cells with LPS and *Il*‐4 to induce the M1 and M2 phenotypes, respectively. M1 macrophages are also called classically activated, proinflammatory macrophages that are involved in the early stage of AKI, whereas M2 macrophages perform profibrotic function in CKD [24, 25]. We found that the levels of miR‐382 were reduced in LPS‐treated macrophages and increased in *Il*‐4‐treated macrophages (Figure 1e). We also evaluated the abundance of miR‐382 in renal macrophages, which were identified as CD45^+^F4/80^+^CD11b^+^ cells and were isolated from mouse kidneys by flow cytometry. Gating strategy for renal macrophage analysis was shown in Figure S1. Interestingly, we found that the miR‐382 expression levels were significantly lower in renal macrophages at 6 and 24 h in mice with IRI compared to those which underwent sham surgery (Figure 1f). Altogether, these results demonstrated the reduction of miR‐382 expression levels after acute injury in both cultured macrophages and primary renal macrophages isolated from kidneys.

### 3.2. Downregulation of miR‐382 Aggravates Oxidative Stress and Apoptosis in mTECs

To explore the role of miR‐382 in oxidative stress and apoptosis, we overexpressed miR‐382 by transfecting mTECs with a miR‐382 mimic. We evaluated oxidative stress by measuring ROS levels and apoptosis by TUNEL staining. We found that the ROS levels and the proportion of TUNEL^+^ cells were significantly higher in mTECs exposed to H/R than in control cultures. However, miR‐382 overexpression alleviated H/R‐induced oxidative stress and apoptosis in miR‐382‐overexpressing mTECs compared to those transfected with the negative control mimic (Figure 2a,b). To confirm the role of miR‐382 in this model, we knocked down miR‐382 by transfecting mTECs with the locked nucleic acid‐modified anti‐miR‐382 and determined that miR‐382 knockdown significantly impaired cell viability induced by H/R compared to the scramble control (Figure 2c). These results indicated that miR‐382 was protective against oxidative stress and apoptosis after AKI in TECs.

Figure 2Downregluation of miR‐382 aggravates oxidative stress and apoptosis in mTECs. (a) Oxidative stress in mTECs was evaluated by ROS detection. ROS was stained as green and nucleic was blue. Compared with ctrl group, mean fluorescence intensity (MFI) of ROS was increased after H/R for 24 h. Compared with NC + H/R group, MFI of ROS was inhibited significantly in mimic + H/R group. (b) Rate of apoptosis cells was stained with TUNEL. Five microscopical fields (200X) were randomly selected per section, and the average TUNEL + cells was calculated. Compared with ctrl group, rate of apoptosis cells elevated in H/R group. Compared with NC + H/R group, rate of apoptosis cells decreased in mimic + H/R group. (c) Cell viability was detected by CCK8 assay. Compared with 21% O_2_ group, cell viability was inhibited in 1% O_2_ group. Compared with 1% O_2_ + anti‐scramble group, cell viability was further hindered in 1% O_2_ + anti‐miR‐382 group. Scale bars = 100 and 200 μm, respectively. Data are presented as means ± SEM; *N* = 4.  ^∗^ *p* < 0.05;  ^∗∗^ *p* < 0.01. The experiments were replicated at least twice.

**Figure:**
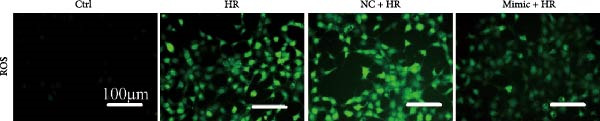
(a)

**Figure:**
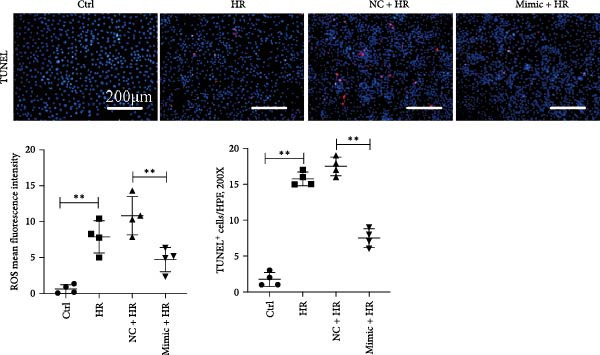
(b)

**Figure:**
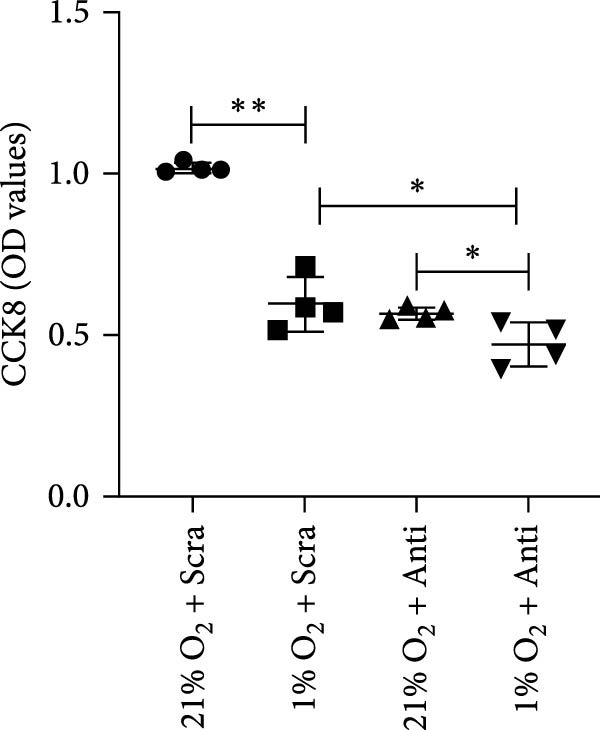
(c)

### 3.3. The STAT1 Pathway Is Activated After AKI In Vivo and In Vitro

STAT1 is highly expressed in proximal tubules, and its activation in TECs has been implicated in various kidney diseases [26–28]. Based on previous studies showing that miR‐382 inhibits STAT1 activation in BPD and pancreatic cancer [20, 21], we determined the regulation of STAT1 by measuring the protein levels of p‐STAT1 S727 and total STAT1 with immunoblotting after AKI both in vivo and in vitro. In vitro, the protein levels of both p‐STAT1 and STAT1 gradually increased in mTECs exposed to H/R for 6, 12, and 24 h, compared to control cultures; the p‐STAT1/STAT1 ratio also exhibited a gradual increase over time (Figure 3a). Additionally, the p‐STAT1/STAT1 ratio was significantly higher in mTECs treated with LPS for 3, 6, 12, and 24 h, compared to control cultures (Figure 3b). In vivo, the protein levels of p‐STAT1 and STAT1 in renal tissue were increased at 12, 24, and 48 h after IRI (Figure 3c). These results demonstrated that STAT1 phosphorylation was accompanied by the progression of AKI.

Figure 3Activation of STAT1 pathway after AKI both in vivo and in vitro. (a) Western blot images for p‐STAT1 S727 and STAT1 in mTECs with H/R treatment for 6, 12, and 24 h, NS as control group. Ratio of p‐STAT1 S727/STAT1 represented degree of phosphorylation of STAT1. Ratio of p‐STAT1 S727/STAT1 increased gradually after H/R induction. (b) Ratio of p‐STAT1 S727/STAT1 also enhanced significantly in mTECs challenged with LPS (5 μg/mL) for 3, 6, 12, and 24 h. (c) AKI was performed with IRI model and protein levels of p‐STAT1 S727 and STAT1 increased significantly in renal of I/R for 12, 24, and 48 h when compared with sham operation. *β*‐actin served as a standard for normalization. Data are presented as means ± SEM; *N* = 4.  ^∗^ *p* < 0.05;  ^∗∗^ *p* < 0.01. The experiments were replicated at least twice.

**Figure:**
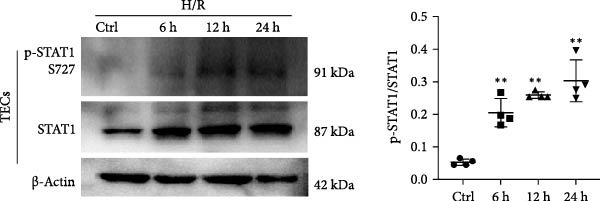
(a)

**Figure:**
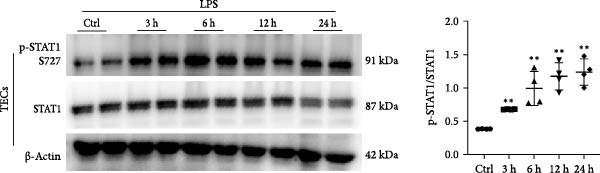
(b)

**Figure:**
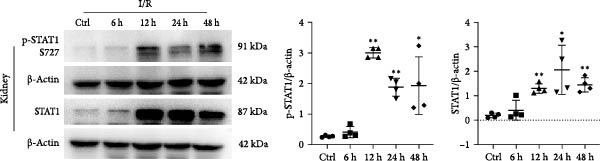
(c)

### 3.4. MiR‐382 Is Involved in the Regulation of STAT1 Signaling in AKI

To determine the relationship between miR‐382 and STAT1 in vitro, we overexpressed miR‐382 in mTECs before exposure to H/R. We found that the protein levels of both p‐STAT1 and STAT1 were increased following H/R exposure; however, this effect was inhibited in cultures overexpressing miR‐382, suggesting that miR‐382 inhibited the activation of STAT1 in mTECs (Figure 4a). We also investigated the relationship of miR‐382 and STAT1 in Raw264.7 cells transfected with the miR‐382 mimic. Immunofluorescence staining indicated that the increased p‐STAT1 levels observed after LPS induction in Raw264.7 cells were abrogated in those overexpressing miR‐382 (Figure 4b). In addition, the p‐STAT1/STAT1 ratio, which increased in Raw264.7 cells following LPS treatment, was abrogated in cultures overexpressing miR‐382 (Figure 4c). Furthermore, the mRNA levels of *Il-6* and *iNos*, which were increased after LPS treatment, were inhibited by miR‐382 overexpression (Figure 4d, e). Altogether, these results provided evidence for the reciprocal and inverse relationship between miR‐382 and STAT1 signaling both in mTECs and Raw264.7 cells.

Figure 4MiR‐382 involved in AKI via STAT1 signaling. (a) Western blot images for p‐STAT1 S727 and STAT1 in mTECs among 21% O_2_ + NC, 21% O_2_ + mimic, 1% O_2_ + NC, and 21% O_2_ + mimic groups, which indicated the reciprocal suppression relationship between miR‐382 and STAT1. Compared with 21% O_2_ + NC, both p‐STAT1 S727 and total STAT1 increased significantly in 1% O_2_ + NC group. Compared with 1% O_2_ + NC, both p‐STAT1 S727 and total STAT1 decreased significantly in 1%O_2_ + mimic group. GAPDH served as a standard for normalization. (b) Immunofluorescence images (1200X) for p‐STAT1 in Raw264.7 among control, LPS (1 μg/mL for 24 h), NC + LPS, and mimic + LPS. p‐STAT1 was stained as red and nucleic was blue. Fluorescence intensity was enhanced after LPS treatment, compared with control group. Fluorescence intensity was inhibited in mimic + LPS group, compared with NC + LPS group. (c) Western blot images for p‐STAT1 S727 and STAT1 in Raw264.7 among NC, mimics, NC + LPS, and mimic + LPS groups. Compared with NC group, ratio of p‐STAT1 S727/STAT1 increased in NC + LPS group. Compared with NC + LPS group, ratio of p‐STAT1 S727/STAT1 was inhibited in mimic + LPS group. *β*‐Actin served as a standard for normalization. (d, e) Quantification of relative mRNA levels of *Il-6* and *iNOS* in Raw264.7 among control, LPS (1 μg/mL for 24 h), NC + LPS, and mimic + LPS. Compared with NC + LPS group, relative mRNA of *Il-6* and *iNOS* were increased significantly in NC + LPS group. Compared with NC + LPS group, relative mRNA of *Il-6* and *iNOS* were inhibited in mimic + LPS group. *β*‐Actin served as standard for normalization (*n* = 4 in each group). Data are presented as means ± SEM; *N* = 4.  ^∗^ *p* < 0.05;  ^∗∗^ *p* < 0.01. The experiments were replicated at least twice.

**Figure:**
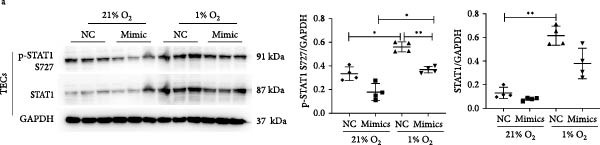
(a)

**Figure:**
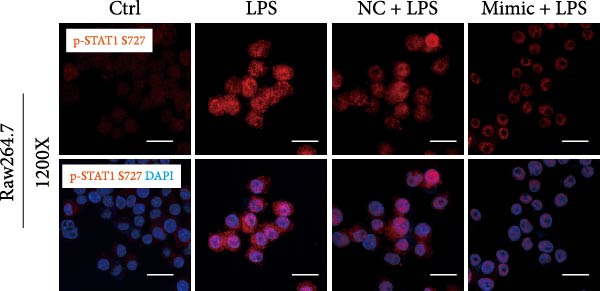
(b)

**Figure:**
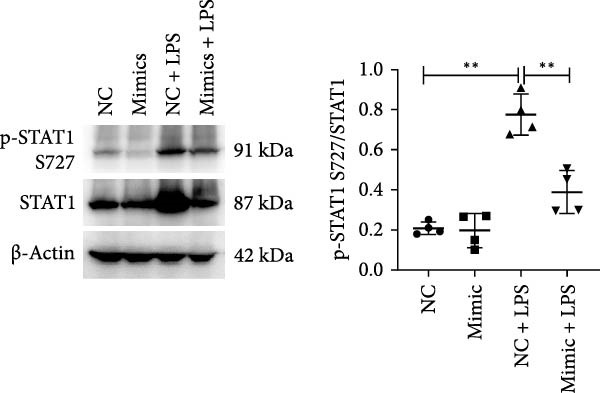
(c)

**Figure:**
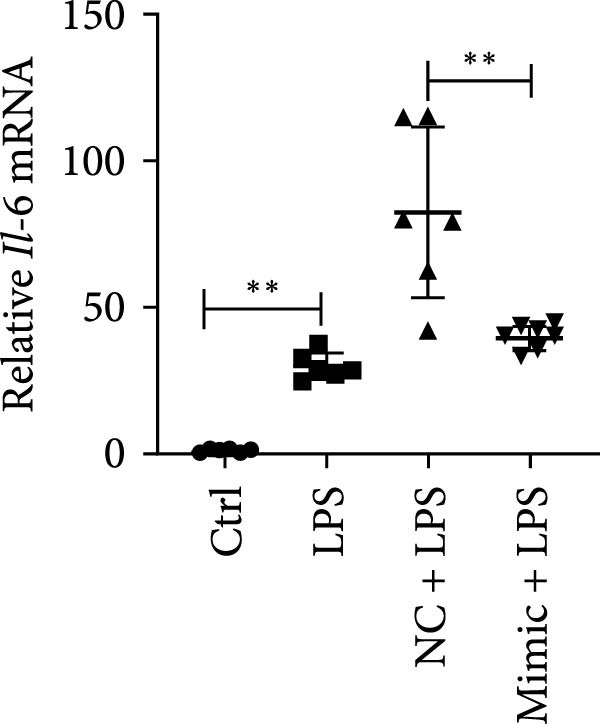
(d)

**Figure:**
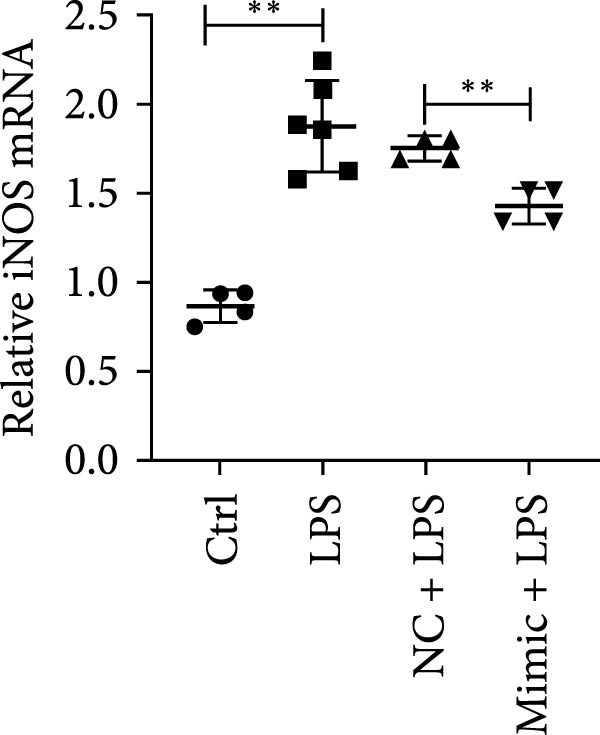
(e)

To further explore the role of STAT1 in LPS‐induced AKI, we inhibited STAT1 activity with fludarabine treatment prior to LPS or H/R exposure in mTECs and prior to LPS treatment in Raw264.7 cells. The appropriate fludarabine concentration was screened in TEC and raw cells separately (Figure S2). We found that the mRNA levels of the proinflammatory cytokine *Il-6* were higher in LPS‐treated mTECs than in control cultures and that this increase was abrogated in mTECs treated with fludarabine (Figure 5a). Furthermore, the mRNA levels of *Tnf-α* and *Il-1β*, which were increased with H/R exposure, were abrogated with STAT1 inhibition in mTECs (Figure 5b, c). To investigate the regulation of STAT1 in macrophages, we treated Raw264.7 cells with fludarabine prior to LPS induction, which significantly inhibited LPS‐induced expression of *iNos* and *Tnf-α* while also increasing the mRNA levels of the anti‐inflammatory cytokine *Il-10* in Raw264.7 cells (Figure 5d–f).

Figure 5Inhibition of STAT1 alleviated H/R‐induced inflammation in mTECs and LPS‐induced M1 polarization in Raw264.7. STAT1 activation was inhibited by treating cells with 5 μM fludarabine 12 h before other treatments. (a) Quantification of relative mRNA levels of *Il-6* in mTECs among control, LPS, DMSO + LPS, and Flu + LPS groups. (b, c) Quantification of relative mRNA levels of *TNF-α* and *Il-1β* in mTECs among 21% O_2_ + DMSO, 21% O_2_ + Flu, 1% O_2_ + DMSO, and 1% O_2_ + Flu groups. (d–f) Quantification of relative mRNA levels of *iNOS*, *TNF-α*, and *Il-10* in Raw264.7 among control, LPS, DMSO + LPS, and Flu + LPS groups. *β*‐Actin served as standard for normalization (*n* = 4 in each group). Data are presented as means ± SEM; *N* = 4.  ^∗^ *p* < 0.05;  ^∗∗^ *p* < 0.01. The experiments were replicated at least twice.

**Figure:**
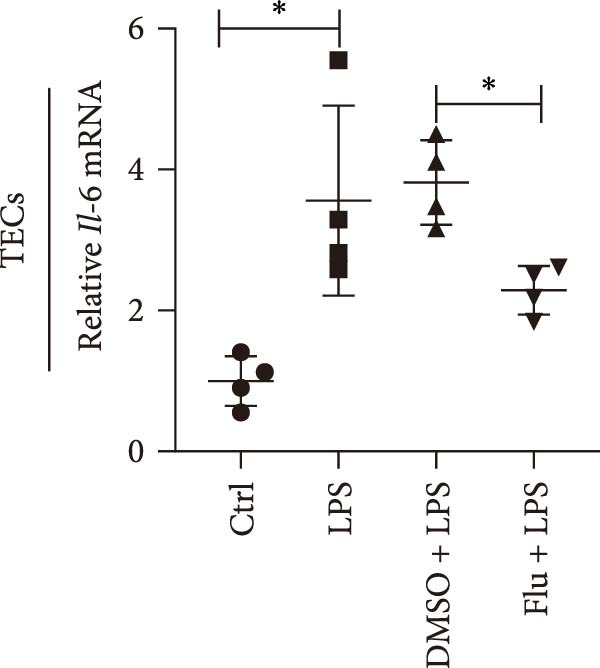
(a)

**Figure:**
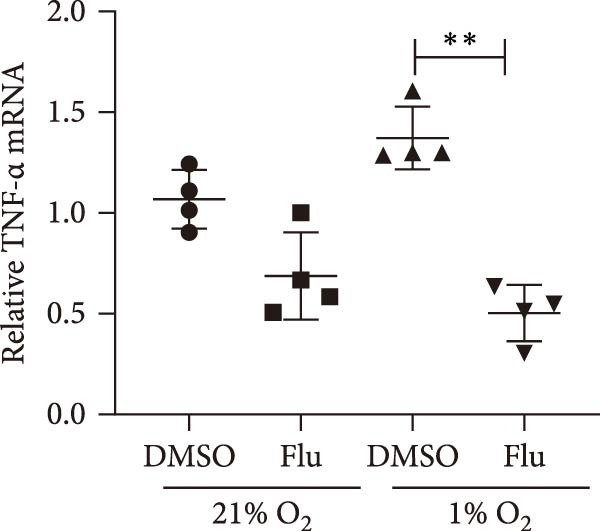
(b)

**Figure:**
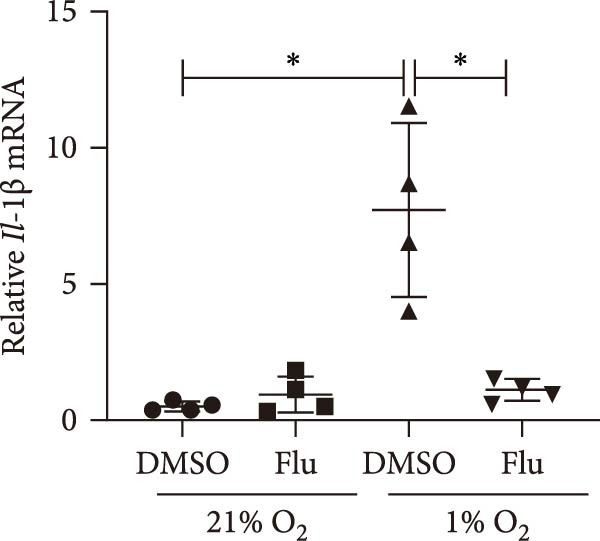
(c)

**Figure:**
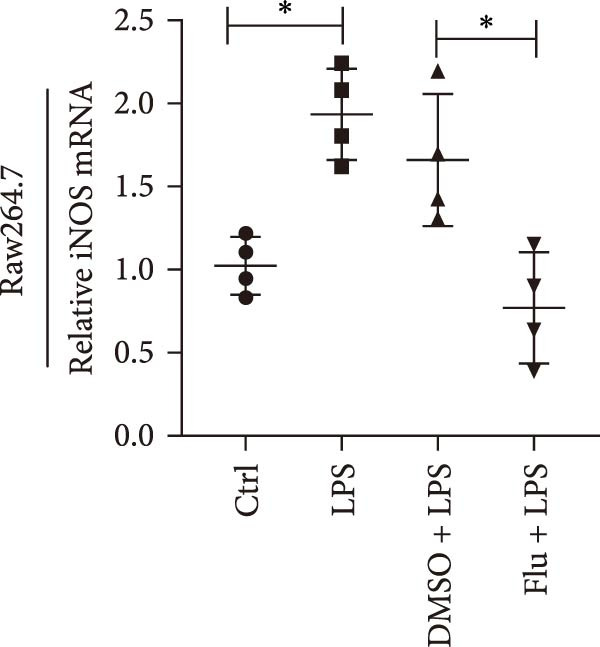
(d)

**Figure:**
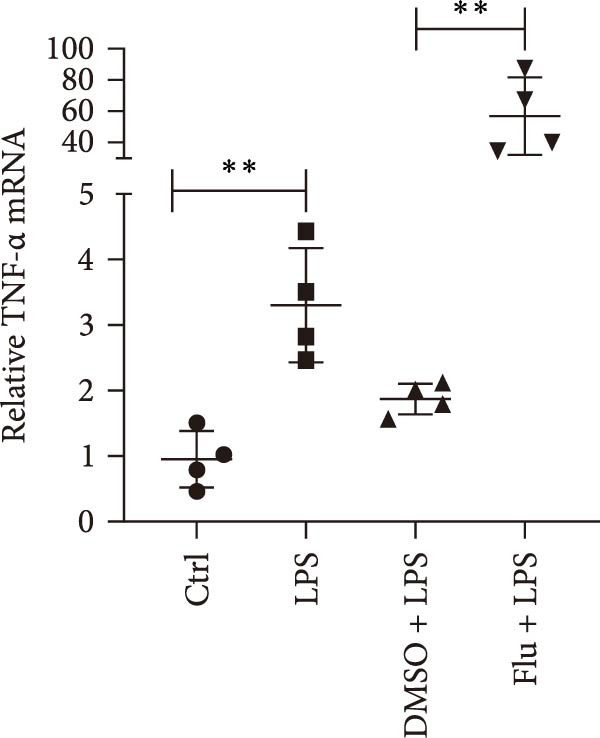
(e)

**Figure:**
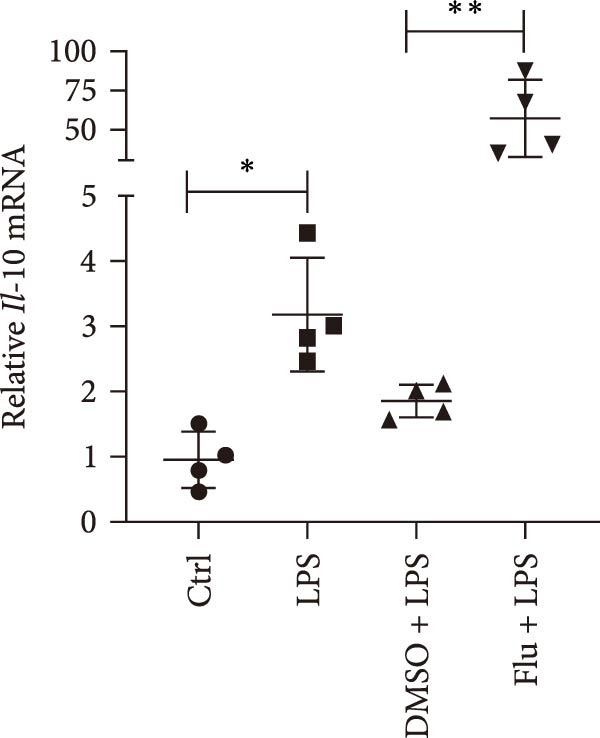
(f)

### 3.5. Knockout of miR‐382 Aggravates IRI‐ and LPS‐Induced AKI In Vivo

Next, we determined the role of miR‐382 in AKI by inducing IRI in miR‐382 knockout mice. The sequence information for the knockout fragment of miR‐382 in knockout mice was provided in Table S1. In situ hybridization for miR‐382 was performed in kidney tissue from miR‐382^−/−^ knockout and wildtype mice (Figure S3a). We performed a time course with IRI for 2, 4, 6, and 24 h and found that the mRNA levels of *Il-1β*, *Il-6*, and *Il-10* significantly increased at all evaluated time points. We also found that the mRNA levels of monocyte chemoattractant protein‐1 (*Ccl-2*) increased after 6 h of IRI while the mRNA levels of *Tnf-α* remained elevated until the 24‐h time point (Figure S4a–e). Therefore, we chose 24 h of IRI for subsequent experiments. We confirmed the efficiency of miR‐382 knockout in kidneys by RT‐qPCR and performed genetic identification using tail samples (Figure 6a). Hematoxylin/eosin staining revealed severe necrosis in tubules and the formation of urinary casts inside the tubules after IRI, and the observed damage was exacerbated by miR‐382 knockout (Figure 6b). The serum creatinine levels were significantly increased in mice exposed to 24‐h of IRI, compared to the sham animals. Additionally, miR‐382 knockout exacerbated renal dysfunction in IRI‐induced AKI (Figure 6c). Furthermore, miR‐382 knockout induced significant upregulation of *Mcp-1* (*Ccl-2*) and *Il-1β* mRNA levels (Figure 6d–e) and exacerbated apoptosis in kidneys (Figure S5a).

Figure 6Knockout of miR‐382 aggravated I/R and LPS induced AKI in mice. (a) MiR‐382^−/−^ mice were bred in the BIORAY Lab, from C57BL/6 J mice back‐crossed to miR‐382^−/−^ mice. Relative miR‐382 expression in the kidneys by RT‐qPCR was examined between wildtype and miR‐382 knockout mice. U6 served as a standard for normalization. Genetic identification was performed with mice tail snip. HE represents heterozygote; WT as wildtype; HO means homozygote and was identified as miR‐382 knockout mice. (b) Representative images of H&E staining in WT‐sham, KO‐sham, WT‐I/R 24 h, and KO‐I/R 24 h groups. H&E staining revealed severe necrosis in the tubules and formation of urinary casts inside the tubules after I/R. Tubular damage score in renal cortical tissues was evaluated. Scale bars = 100 μm. (c) Renal function was evaluated by serum creatinine. Compared with sham operation, level of serum creatinine elevated notably after I/R. Compared with WT‐I/R group, serum creatinine was further increased in KO‐I/R group. (d–e) Quantification of relative mRNA levels of *Ccl-2* and *Il-1β* in renal tissue among WT‐Sham, KO‐Sham, WT‐I/R, and KO‐I/R groups. *β*‐Actin served as standard for normalization. (f) Sepsis‐induced AKI was induced by a single intraperitoneal injection of 8 mg/kg of LPS. Tissue ROS production was detected on frozen kidney sections using dihydroethidium (DHE) staining. Representative images of DHE staining were shown between WT‐LPS and KO‐LPS groups. We randomly choose five microscopical field per sample. MFI of DHE was calculated among WT‐NS, KO‐NS, WT‐LPS, and KO‐LPS groups. (*n* = 6 in each group). Scar bar = 200 μm. (g–h) Quantification of relative mRNA levels of *NGAL* and *Il-6* in renal tissue among WT‐NS, KO‐NS, WT‐LPS, and KO‐LPS groups. *β*‐Actin served as standard for normalization. Data are presented as means ± SEM; *N* = 4.  ^∗^ *p* < 0.05;  ^∗∗^ *p* < 0.01. The experiments were replicated at least twice.

**Figure:**
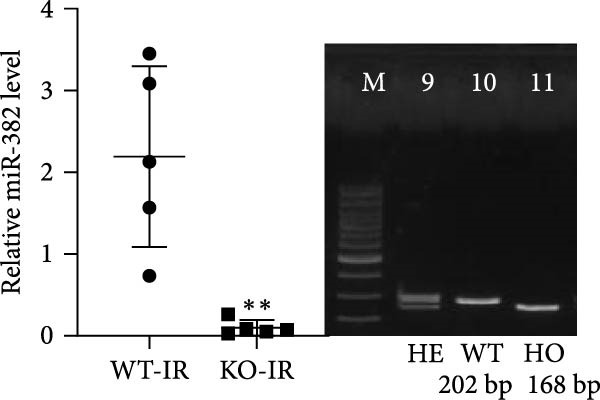
(a)

**Figure:**
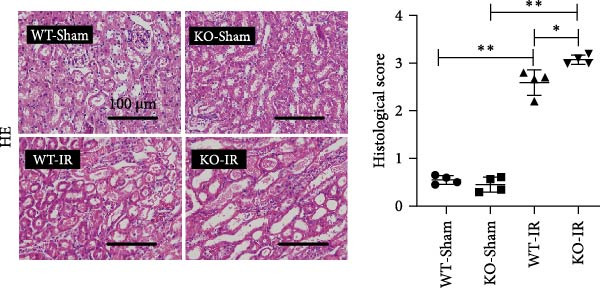
(b)

**Figure:**
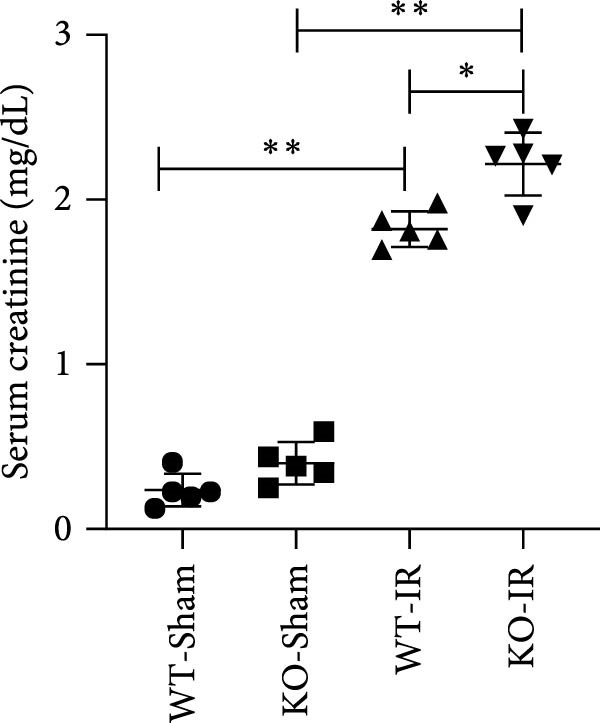
(c)

**Figure:**
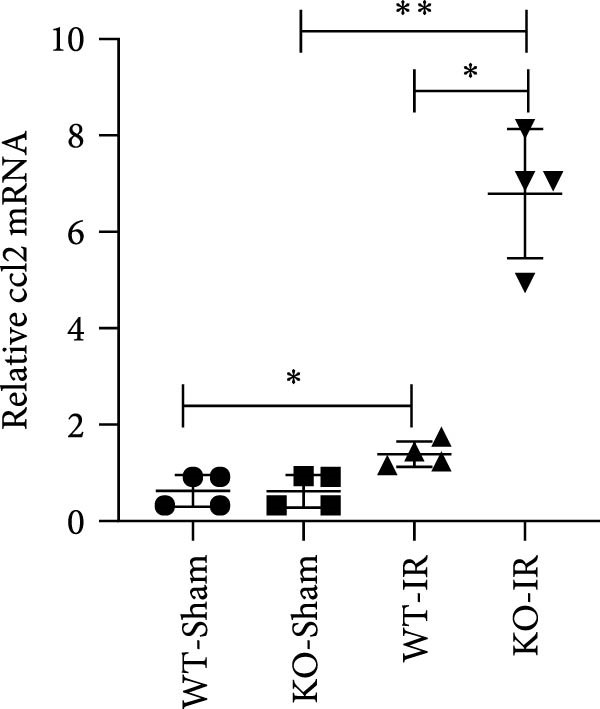
(d)

**Figure:**
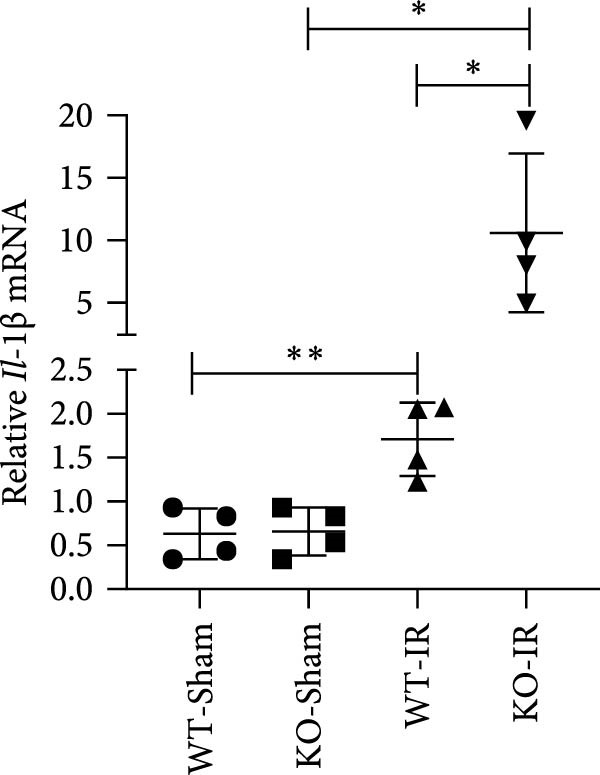
(e)

**Figure:**
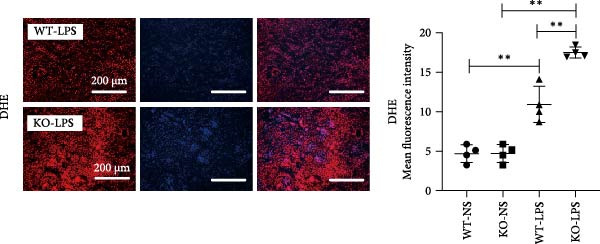
(f)

**Figure:**
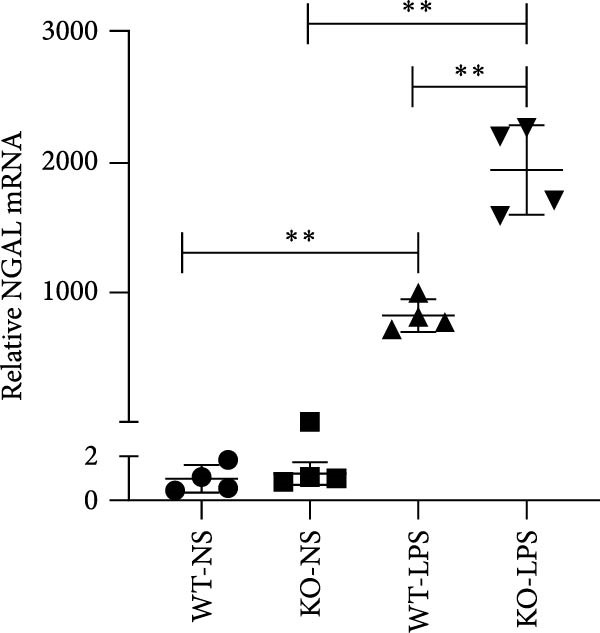
(g)

**Figure:**
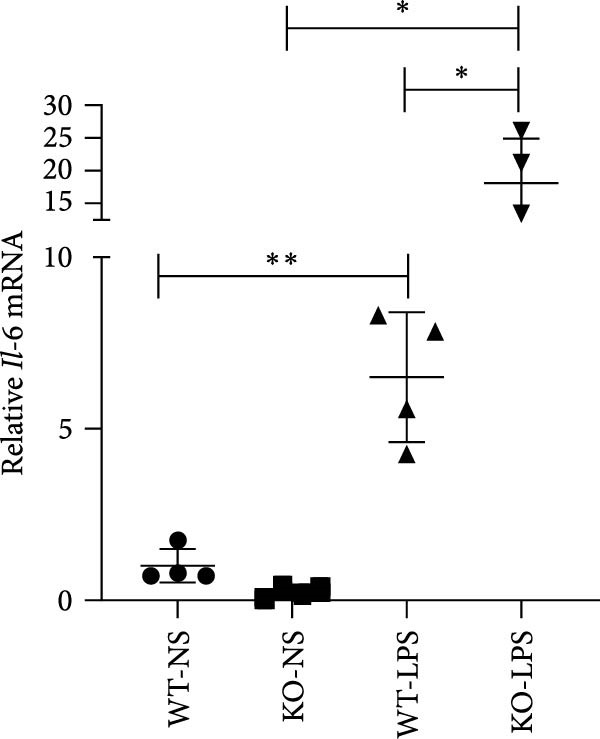
(h)

We also examined the role of miR‐382 in AKI by evaluating miR‐382 knockout mice challenged by LPS administration. Hematoxylin/eosin staining revealed cell vacuolization, tubular necrosis, and loss of the brush border in kidneys of LPS‐treated mice. In contrast, the morphologic changes in kidneys were more severe in miR‐382 knockout mice (Figure S6a). Immunohistochemical staining for anti‐F4/80 revealed increased macrophage infiltration in renal sections following LPS treatment whereas miR‐382 knockout augmented these findings compared to the wildtype mice (Figure S6b). Dihydroethidium staining to assess ROS production following LPS treatment indicated that ROS production was higher in miR‐382 knockout mice than in the wildtype mice (Figure 6f). NGAL is a marker of AKI [29]. The mRNA levels of *Ngal* and *Il-6* were higher in miR‐382 knockout mice than in wildtype mice in the LPS‐induced AKI model (Figure 6g, h). Overall, these data suggested that the knockout of miR‐382 aggravated I/R‐induced and LPS‐induced AKI through the activation of oxidative stress, apoptosis, and inflammatory response, consistent with our in vitro findings.

### 3.6. Knockout of miR‐382 Promotes STAT1 Phosphorylation in IRI‐Induced AKI In Vivo

**Figure 7:**
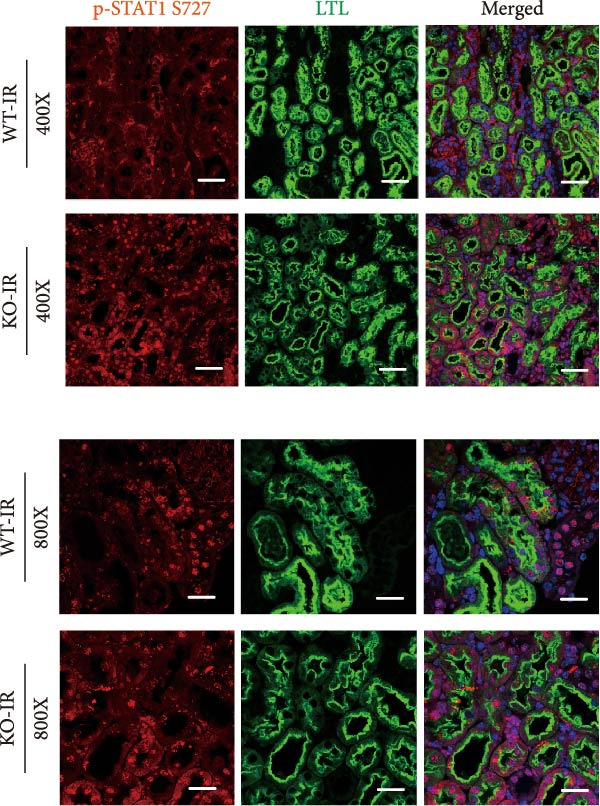
Knockout of miR‐382 promotes phosphorylation of STAT1 in AKI in mice. Co‐staining for p‐STAT, LTL (proximal tubular epithelial marker), and DAPI in renal sections from WT and miR‐382 knockout (KO) mice after 24 h IR treatment. Images were captured at 400X and 800X magnification. Scale bars = 200 and 100 μm, respectively. The experiments were replicated at least twice.

To confirm the activation of STAT1 in proximal tubules, we costained the kidney tissues with p‐STAT1 and the renal proximal tubular marker LTL and found that STAT1 activation occurred primarily in renal PTECs in IRI‐induced AKI in vivo. Moreover, the STAT1 activation in PTECs was higher in miR‐382 knockout mice than in the wildtype mice (Figure 7a).

## 4. Discussion

In the present study, we evaluated the protective role of miR‐382 in I/R‐ and LPS‐induced AKI in vivo and demonstrated that miR‐382 protected against AKI by suppressing inflammation and apoptosis in I/R‐induced AKI. Additionally, miR‐382 exerted a protective effect by reducing inflammation and ROS production in LPS‐induced AKI. The colocalization of p‐STAT1 in PTECs expressing LTL indicated that STAT1 signaling was likely activated in renal PTECs. In vitro, downregulation of miR‐382 in TECs impaired cell viability while promoting apoptosis, oxidative stress, and inflammatory response, possibly through STAT1 signaling. Moreover, the loss of miR‐382 in renal macrophages challenged with LPS led to STAT1 activation and polarization to the M1 phenotype characterized by the upregulation of *Il-6* and *iNos* mRNA levels. Consistently, the loss of miR‐382 was detected in macrophages isolated from kidneys of mice following I/R‐induced AKI. In contrast, miR‐382 was upregulated in M2 macrophages incubated with *Il*‐4 and macrophages isolated from mice in CKD models using unilateral ureteral obstruction (UUO), unilateral I/R (UIR) and aristolochic acid nephropathy (AAN), consistent with our previous findings [30]. This is the first study to demonstrate the critical role of miR‐382 in AKI pathology, which likely occurs through STAT1 activation.

Our study provides a comprehensive analysis of the role of miR‐382 in AKI. Actually, there are similarities and differences in miR‐382’s function in AKI compared to other diseases, such as liver regeneration and fibrosis, tumors, inflammatory metabolic disorders, and renal fibrosis. In liver diseases, miR‐382 has been shown to promote hepatocyte proliferation and liver regeneration after partial hepatectomy by targeting PTEN, thereby activating the PI3K‐AKT pathway [31], which is aligned with our findings in AKI, where miR‐382 enhances cell survival and reduces apoptosis. And miR‐382 displays context‐dependent functions in cancer. In gastric cancer, hypoxia‐induced miR‐382 promotes angiogenesis by suppressing PTEN, facilitating tumor growth [32]. Conversely, in osteosarcoma, miR‐382 acts as a tumor suppressor by inhibiting metastasis and cancer stem cell (CSC) proliferation through YB‐1 targeting [33]. This dichotomy highlights that miR‐382’s role may depend on the cellular microenvironment and downstream signaling pathways, which could also influence its effects in AKI. In BPD, miR‐382‐5p suppresses M1 macrophage polarization and inflammation by targeting CDK8 and inhibiting STAT1 signaling [20]. This anti‐inflammatory mechanism resembles our observations in AKI, where miR‐382 attenuates renal inflammation. In renal fibrosis, miR‐382 has been shown to promote renal fibrosis after UUO induction by targeting kallikrein 5, thereby hindering degradation of extracellular matrix proteins [34]. Such suppression of ECM degradation might play a favorable role during the early stage of AKI.

Our findings also suggest that the miR‐382/STAT1 pathway is involved in the simultaneous regulation of mTECs and macrophage phenotypes. Considerable evidence suggests that both PTECs and renal macrophages play critical roles in kidney diseases including AKI. PTECs are considered the primary target cells and a dominant trigger of kidney diseases as they secrete inflammatory cytokines during interstitial inflammation [35]. Exosomes containing miRNA‐19b‐3p originating from TECs promote M1 macrophage activation in kidney injury [36]. Exosomal *Ccl-2* from TECs is critical for albumin‐induced tubulointerstitial inflammation [37]. Additionally, specific phenotypes of renal macrophages have been shown to contribute to kidney injury and repair [38]. As previously reported, iNOS‐positive proinflammatory, that is, M1, macrophages are recruited to the kidneys in the first 48 h after IRI [38] whereas sirtuin‐6 overexpression promotes M2 macrophages, alleviating renal injury [39]. We demonstrated that miR‐382/STAT1 signaling contributed to the regulation of macrophages in vitro. However, our study has some limitations. We were not able to clarify the relationship between tubular cells and renal macrophages, and it remains unclear which cell type plays a more important role in AKI. More evidence should be provided about the role of miR‐382 in AKI by lentivirus‐mediated miR‐382 overexpression in mice.

In conclusion, our study proposes a novel mechanism wherein downregulation of miR‐382 mediates oxidative stress and apoptosis of tubular cells and the M1 polarization of macrophages in AKI, likely through the activation of STAT1 signaling, thereby providing a promising and novel therapeutic strategy for AKI.

## Conflicts of Interest

The authors declare no conflicts of interest.

## Author Contributions

Xiaoyan Wang, Guo Cheng, and Ting Ren contributed equally to this work.

## Funding

This work was supported by the Science and Technology Commission of Shanghai (Grants 14DZ2260200 and 20DZ2271600) and the National Natural Science Foundation of China Grants 91849123 (to Xiaoqiang Ding) and 82170695 (to Ping Jia).

## Supporting Information

Additional supporting information can be found online in the Supporting Information section.

## Supporting information


**Supporting Information** Figure S1. Gating strategy for renal macrophage. Figure S2. The proper fludarabine concentration was determined both in TECs and Raw264.7 cells. Figure S3. In situ hybridization for miR‐382 in kidney tissue from miR‐382^−/−^ knockout and wildtype mice. Figure S4. Expression of inflammatory cytokines in the time course of I/R. Figure S5. TUNEL staining in renal sections between WT and KO mouse in AKI. Figure S6. H&E and IHC for F4/80 staining in renal between WT and KO mouse by LPS administration. Figure S7. Negative control (NC) antibody staining for p‐STAT1 Ser727 in renal sections and Raw264.7. Table S1. The sequence information for the knockout fragment of miR‐382 in knockout mice. Supporting information: raw data of the western blot studies.

## Data Availability

The figures and table used to support the findings of this study are included within the article and the supporting information file.
