# Supplementary material for: MicroRNA‐382 Is Involved in Acute Kidney Injury via Regulating STAT1 Signaling
Source: J Immunol Res. 2026 Jan 9;2026:5266272. doi: 10.1155/jimr/5266272 (PMC13140935; doi:10.1155/jimr/5266272)
Supplement: Supplementary file 1 — Supporting Information Figure S1. Gating strategy for renal macrophage. Figure S2. The proper fludarabine concentration was determined both in TECs and Raw264.7 cells. Figure S3. In situ hybridization for miR‐382 in kidney tissue from miR‐382−/− knockout and wildtype mice. Figure S4. Expression of inflammatory cytokines in the time course of I/R. Figure S5. TUNEL staining in renal sections between WT and KO mouse in AKI. Figure S6. H&E and IHC for F4/80 staining in renal between WT and KO mouse by LPS administration. Figure S7. Negative control (NC) antibody staining for p‐STAT1 Ser727 in renal sections and Raw264.7. Table S1. The sequence information for the knockout fragment of miR‐382 in knockout mice. Supporting information: raw data of the western blot studies. [file JIMR-2026-5266272-s001.zip › Supplementary Figure.docx]

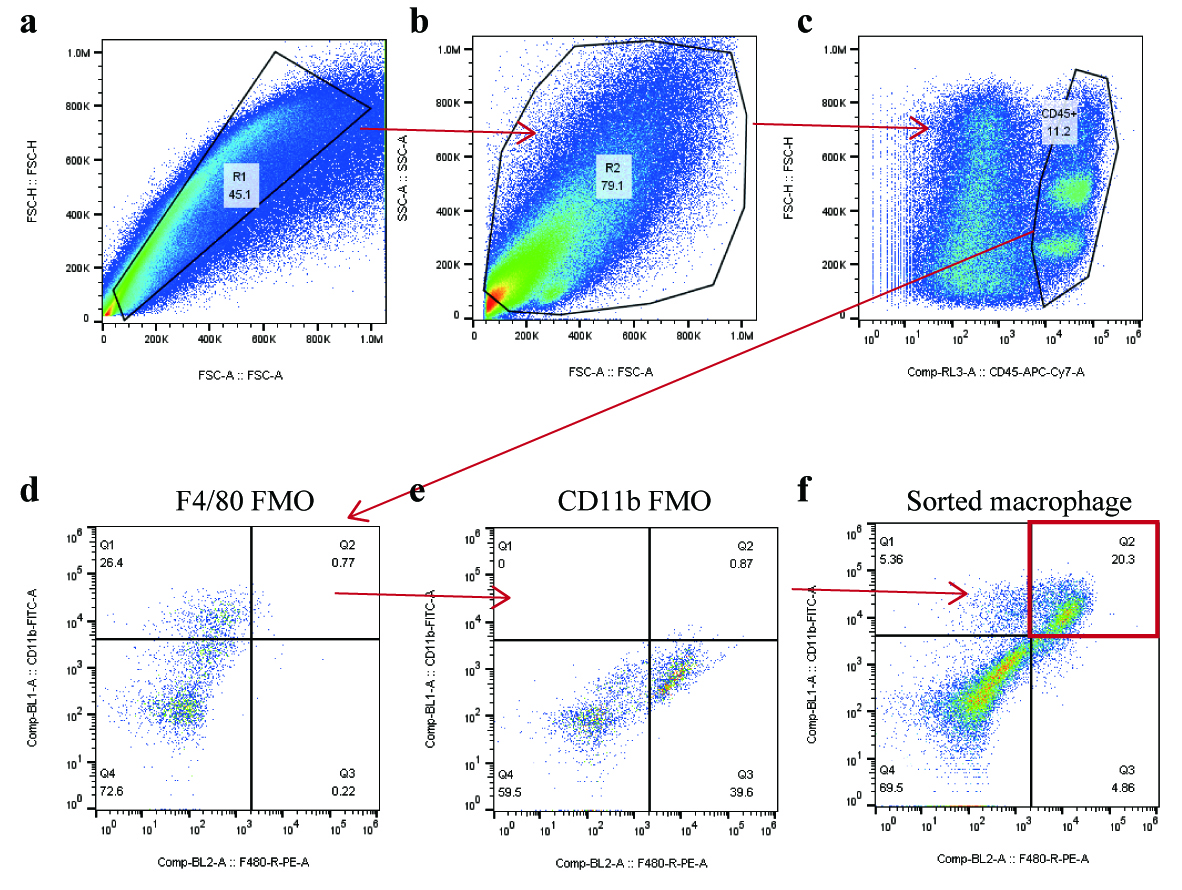


**Supplementary Figure 1**. **Gating strategy for renal macrophage.** The procedure involved a sequential gating strategy: (a-b) Viable single cells were first selected. (c) From this population, CD45^+^ leukocytes were enriched. (d-e) The positive gates for F4/80 and CD11b were demarcated using their respective FMO controls. (f) Macrophages were ultimately defined as F4/80^+^CD11b^+^ cells within the CD45^+^ population.


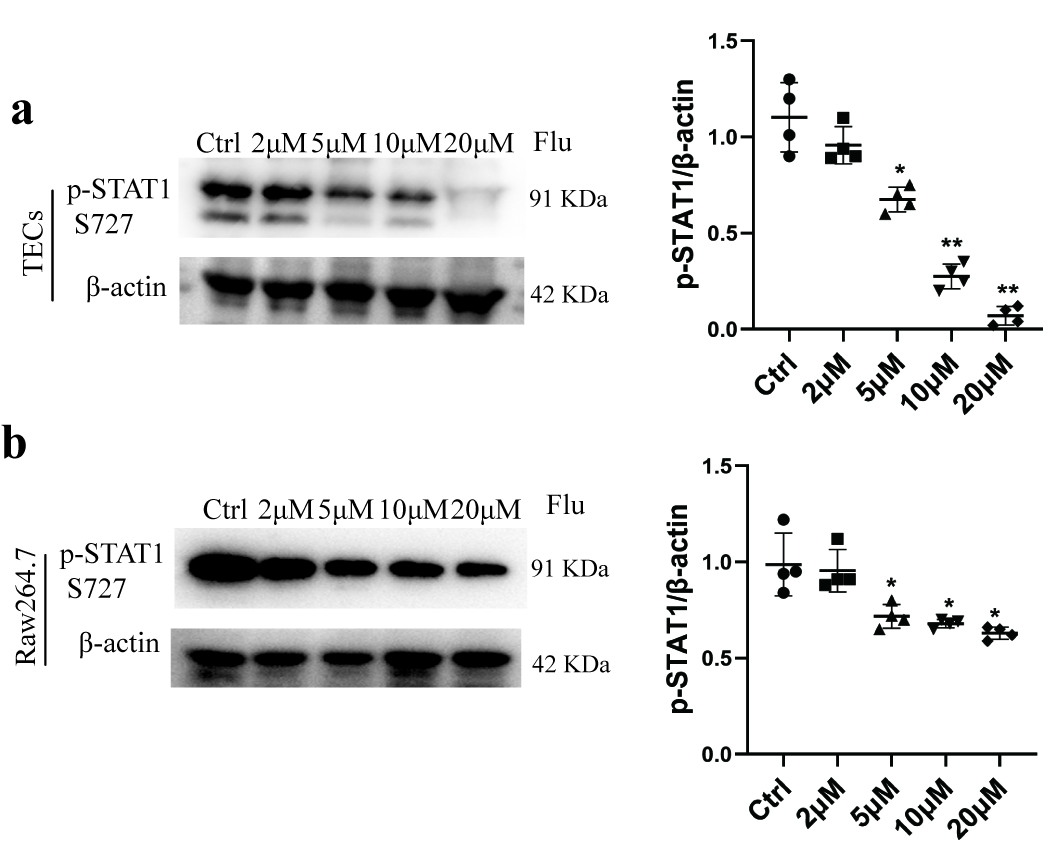


**Supplementary Figure 2. The proper fludarabine concentration was determined both in TECs and Raw264.7 cells.** (a) Western blot images for p-STAT1 S727 in mTECs incubated by 2, 5, 10 and 20μM fludarabine for 24 hours. (b) Western blot images for p-STAT1 S727 in Raw264.7 incubated by 2, 5, 10 and 20μM fludarabine for 24 hours.β-actin served as a standard for normalization. Data are presented as means±SEM; N=4, * *P* < 0.05; ** *P* < 0.01. The experiments were replicated at least twice.


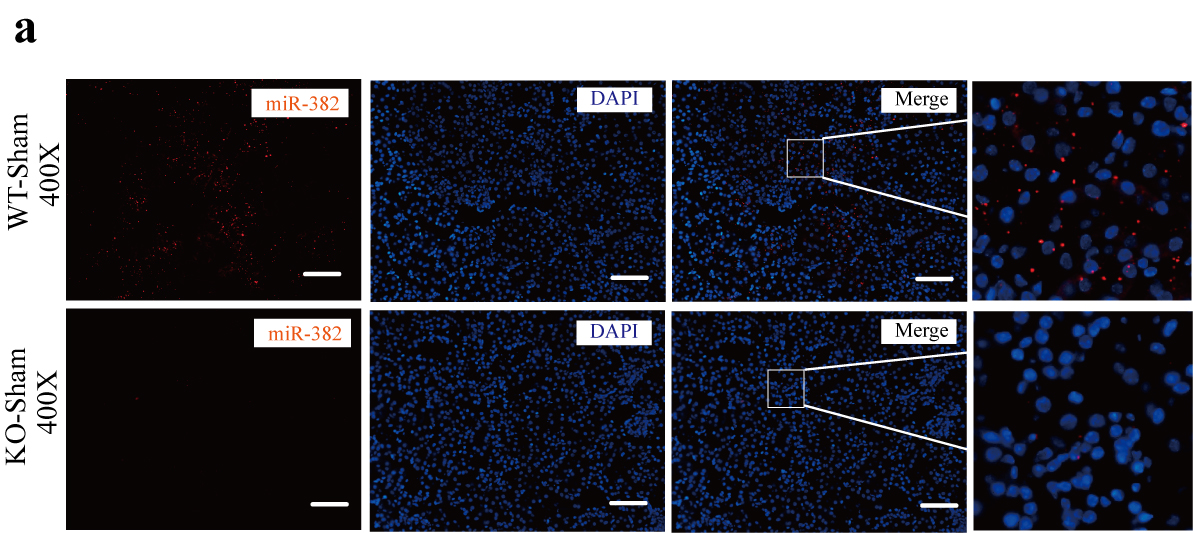


**Supplementary Figure 3. In situ hybridization for miR-382 in kidney tissue from miR-382-/- knockout and wildtype mice.** (a) Representative images of miR-382 FISH in kidney sections between miR-382-/- knockout and wildtype mice. Scale bars = 200 μm. The experiments were replicated at least twice.


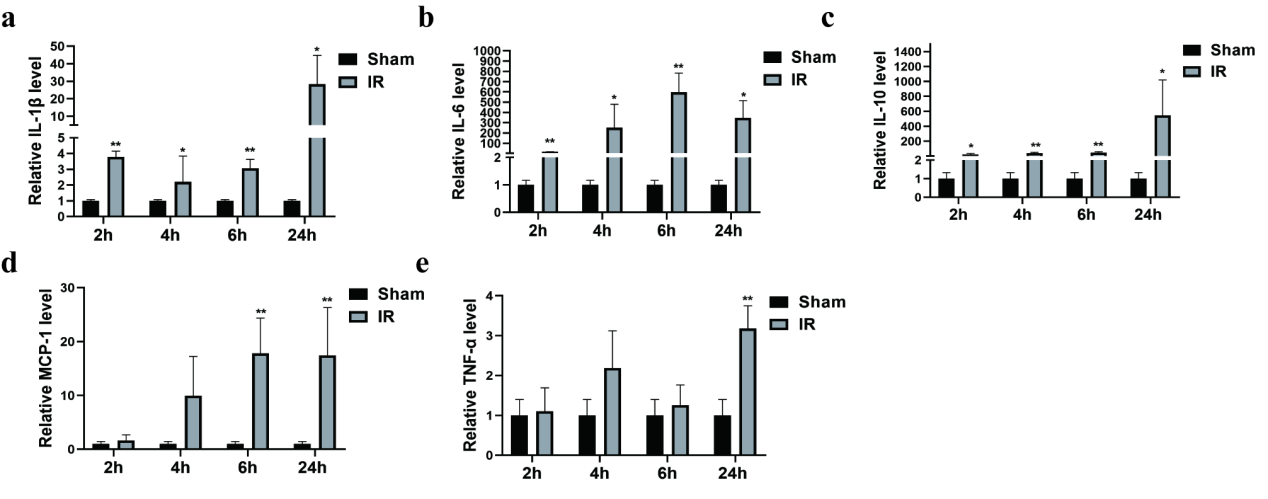


**Supplementary Figure 4. Expression of inflammatory cytokines in the time course of I/R.** (a-e) Quantification of relative mRNA level of *IL-1β, IL-6, IL-10, MCP-1(CCL-2)* and *TNF-α* in kidney after I/R 2, 4, 6 and 24 hours. β-actin served as standard. Data are presented as means±SEM; N=4, * *P* < 0.05; ** *P* < 0.01. The experiments were replicated at least twice.


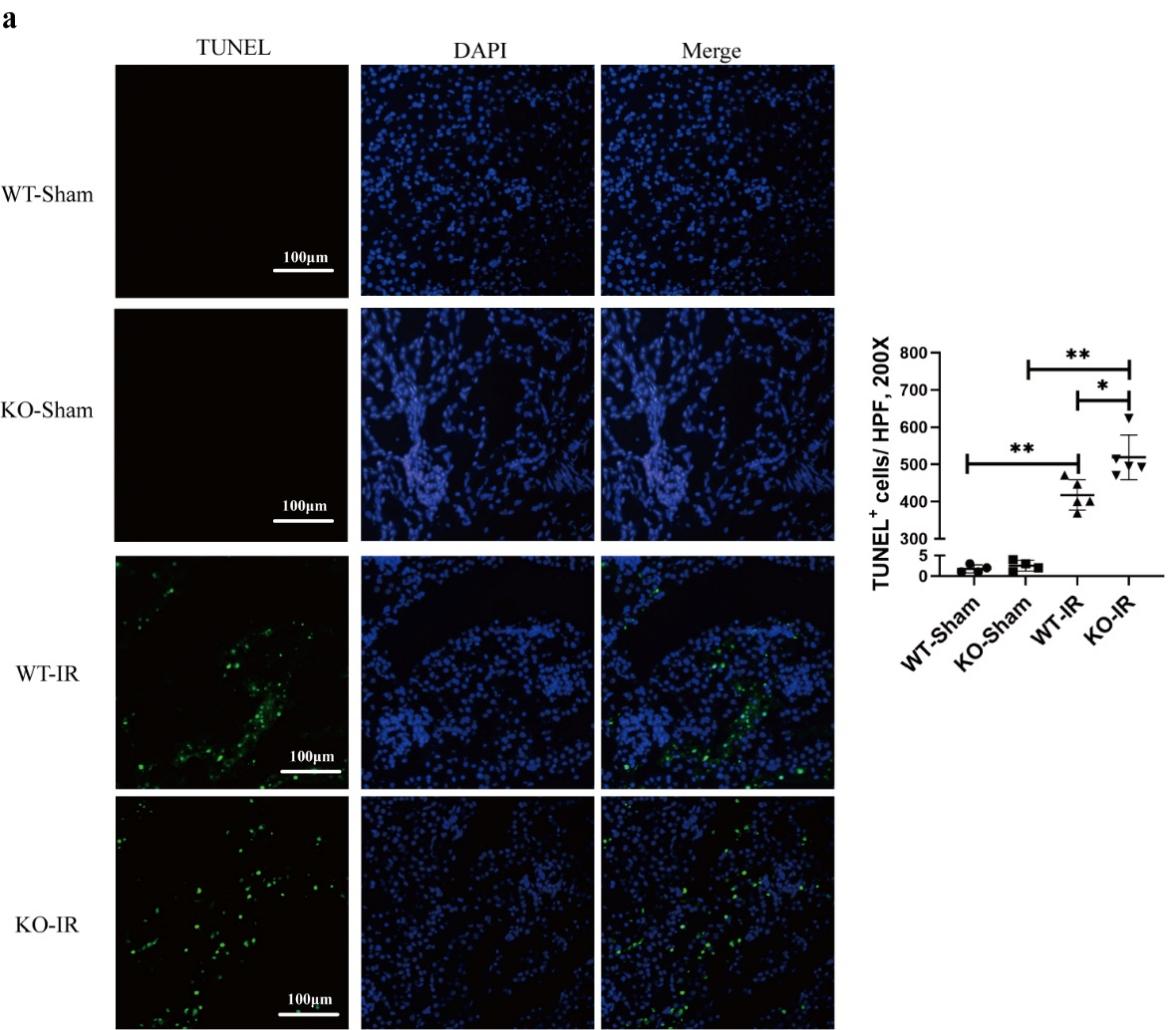


**Supplementary Figure 5. TUNEL staining in renal sections between WT and KO mouse in AKI.** (a-b) Representative images of TUNEL staining in renal sections among WT-sham, KO-sham, WT-I/R 24h and KO-I/R 24h groups. Rate of apoptosis cells was stained with TUNEL. Five microscopical fields (200X) were randomly selected per section, and the average TUNEL+ cells (green) was calculated. Scale bars = 100 μm, respectively. Data are presented as means±SEM; N=4, * *P* < 0.05; ** *P* < 0.01. The experiments were replicated at least twice.


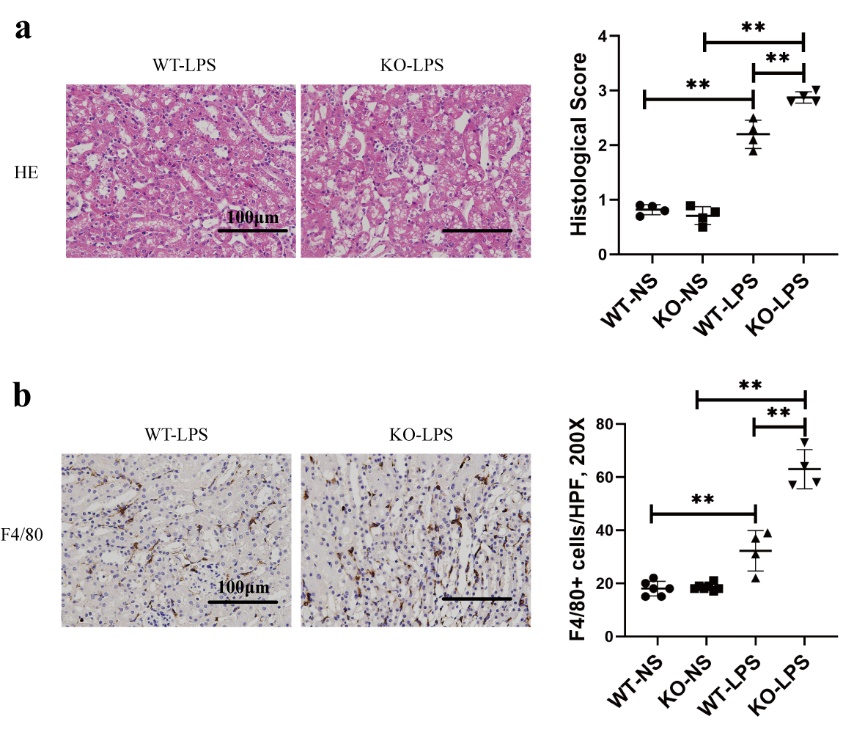


**Supplementary Figure 6. H&E and IHC for F4/80 staining in renal between WT and KO mouse by LPS administration.** (a) Representative images of H&E staining in WT-LPS and KO-LPS groups. Tubular damage score in renal cortical tissues was evaluated. (b) Representative images of IHC staining with anti-F4/80 in WT-LPS and KO-LPS groups. Five microscopical fields (200X) were randomly selected per section, and the average F4/80+ cells were calculated. Scale bars = 100 μm, respectively. Data are presented as means±SEM; N=4, * *P* < 0.05; ** *P* < 0.01. The experiments were replicated at least twice.


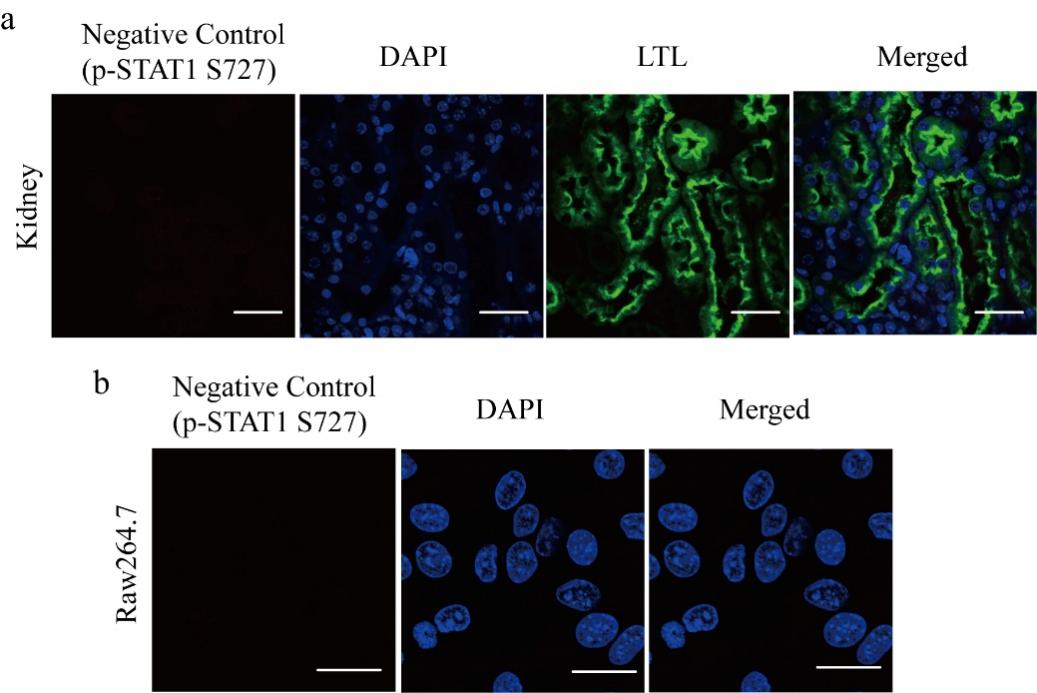


**Supplementary Figure 7. Negative control (NC) antibody staining for p-STAT1 Ser727 in renal sections and Raw264.7** (a) Representative images (400X) of NC antibody staining for p-STAT1 Ser727 in renal sections. (b) Immunofluorescence images (1200X) NC antibody staining for p-STAT1 Ser727 on in Raw264.7.
