# Supplementary figures and images for: MicroRNA‐382 Is Involved in Acute Kidney Injury via Regulating STAT1 Signaling
Source: J Immunol Res. 2026 Jan 9;2026:5266272. doi: 10.1155/jimr/5266272 (PMC13140935; doi:10.1155/jimr/5266272)

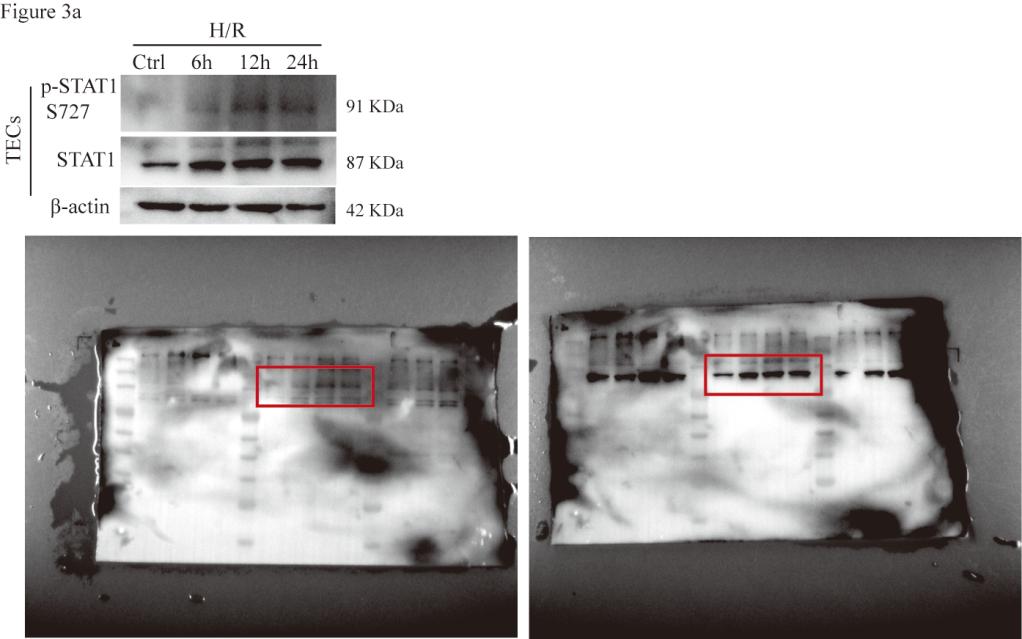


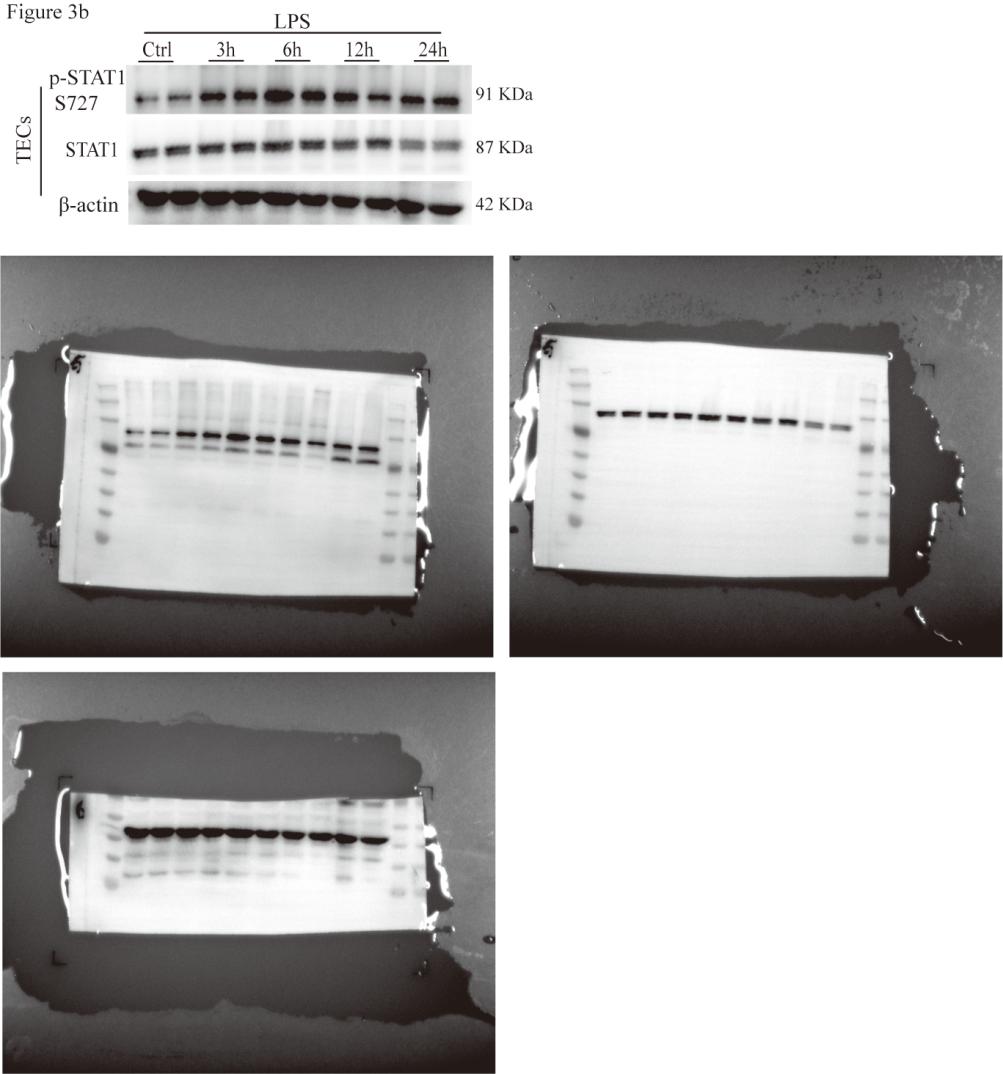


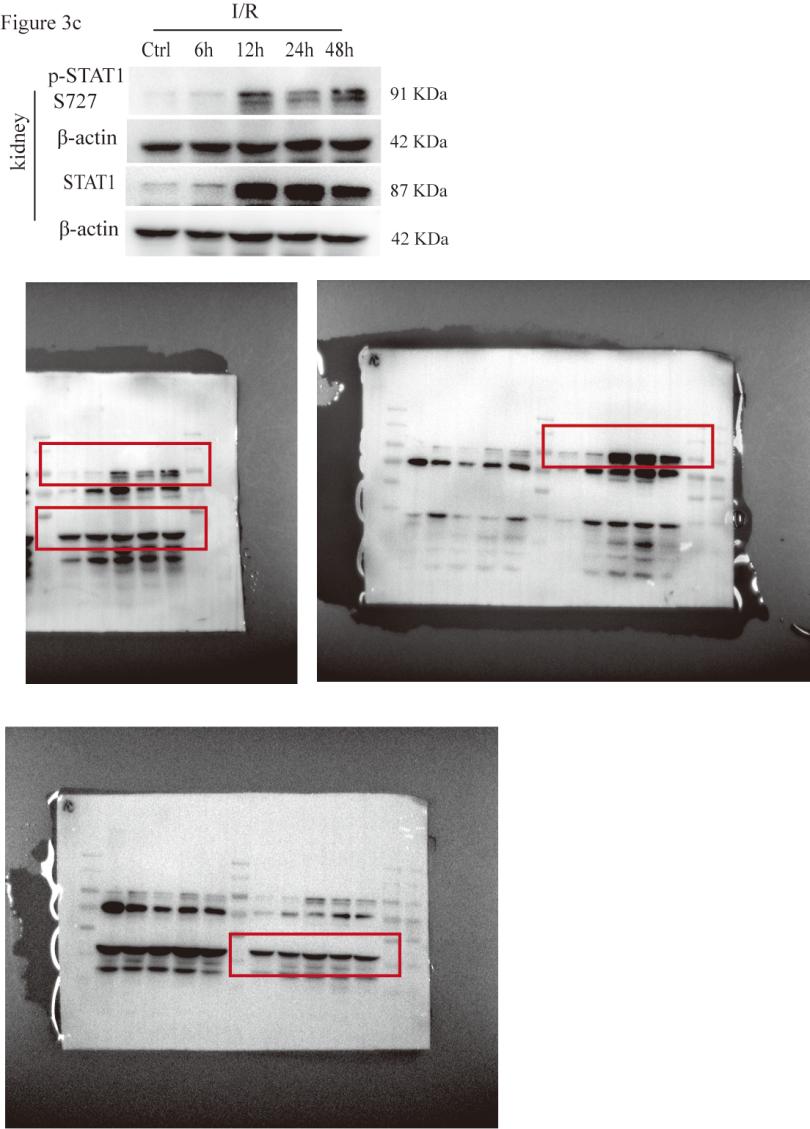


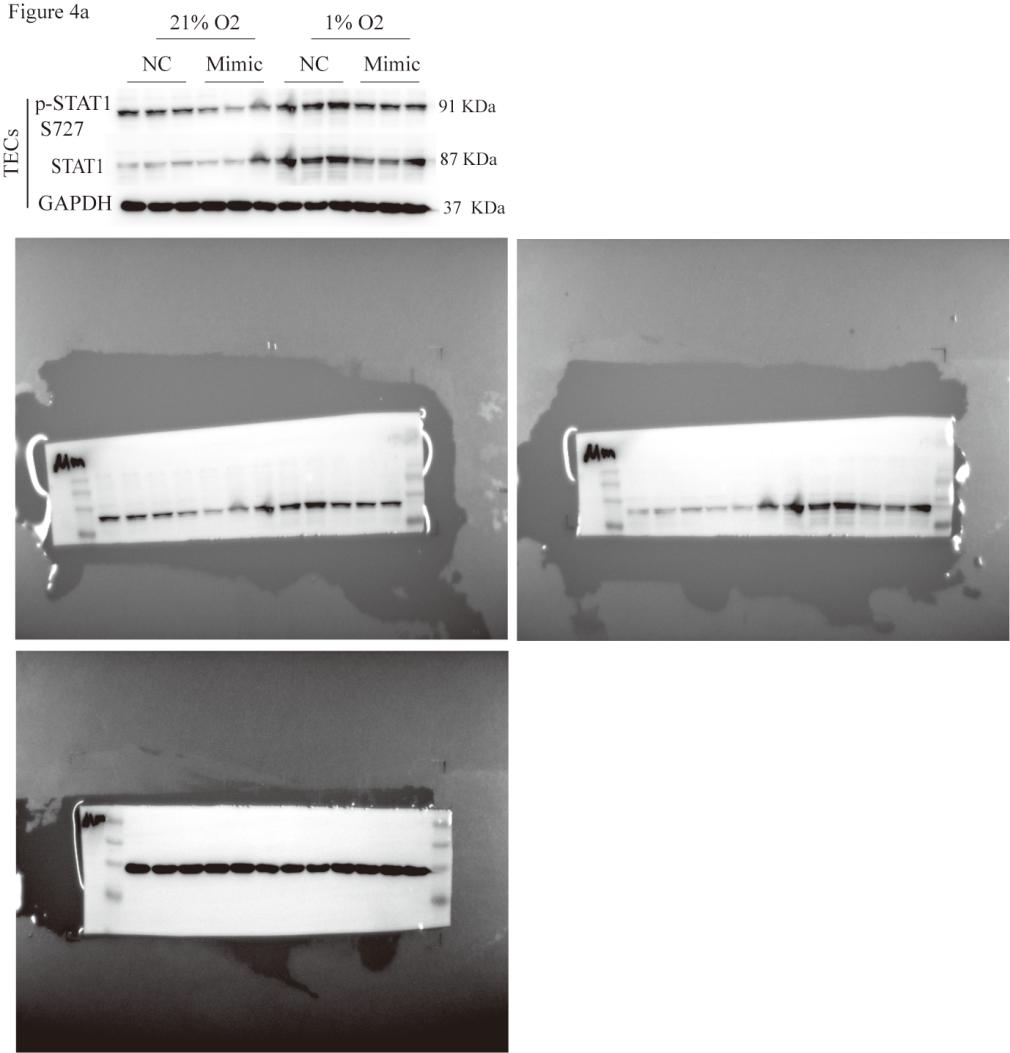


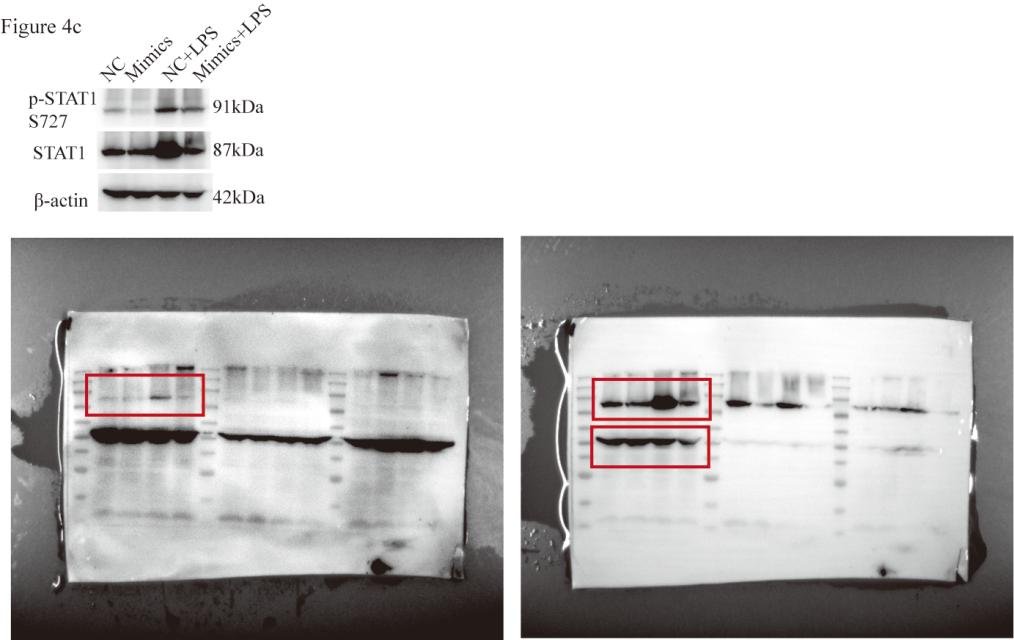


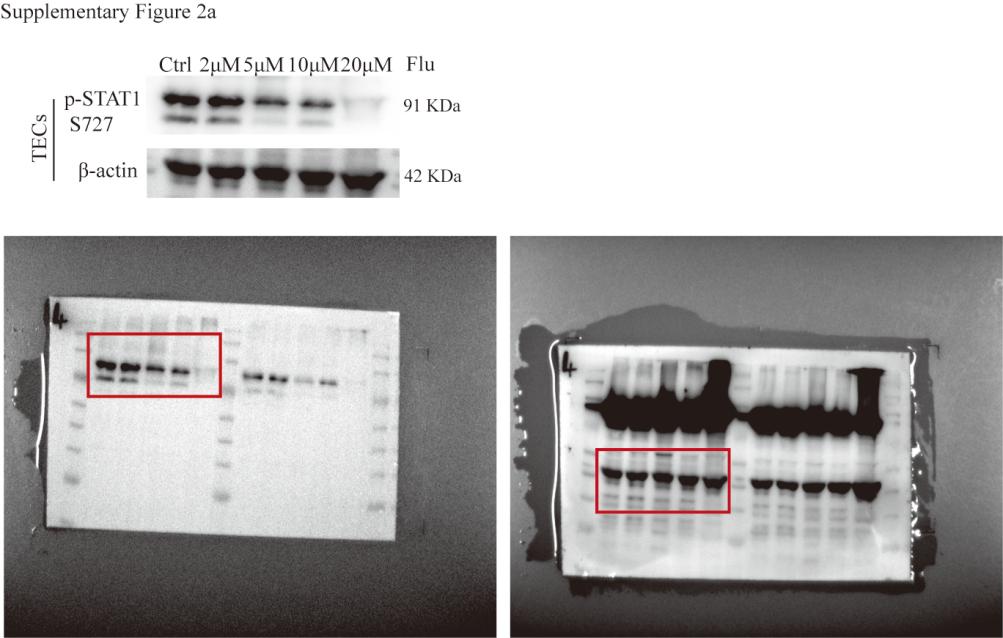


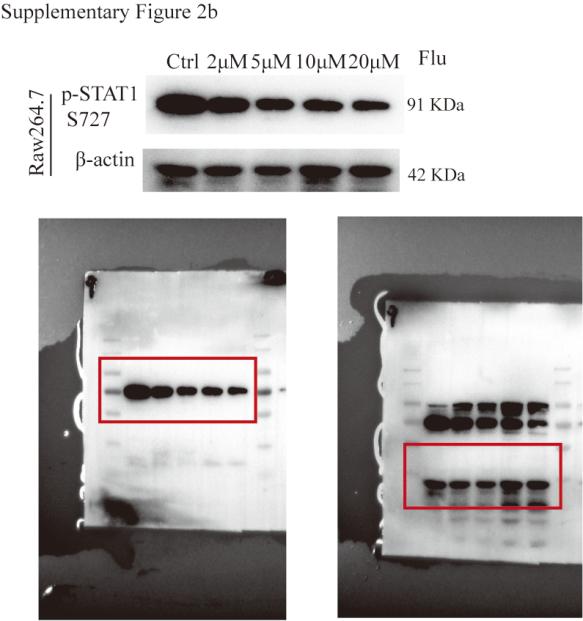

Supplement: Supplementary file 1 — Supporting Information Figure S1. Gating strategy for renal macrophage. Figure S2. The proper fludarabine concentration was determined both in TECs and Raw264.7 cells. Figure S3. In situ hybridization for miR‐382 in kidney tissue from miR‐382−/− knockout and wildtype mice. Figure S4. Expression of inflammatory cytokines in the time course of I/R. Figure S5. TUNEL staining in renal sections between WT and KO mouse in AKI. Figure S6. H&E and IHC for F4/80 staining in renal between WT and KO mouse by LPS administration. Figure S7. Negative control (NC) antibody staining for p‐STAT1 Ser727 in renal sections and Raw264.7. Table S1. The sequence information for the knockout fragment of miR‐382 in knockout mice. Supporting information: raw data of the western blot studies. [file JIMR-2026-5266272-s001.zip › Supplementary material.docx]
